# Behavior of higher-order MDD on energy ratios at the interface of thermoelastic and piezothermoelastic mediums

**DOI:** 10.1038/s41598-023-44339-5

**Published:** 2023-10-11

**Authors:** M. S. Barak, Hijaz Ahmad, Rajesh Kumar, Rajneesh Kumar, Vipin Gupta, Fuad A. Awwad, Emad A. A. Ismail

**Affiliations:** 1 Department of Mathematics, Indira Gandhi University, Meerpur (Rewari), Haryana 123401 India; 2 Section of Mathematics, International Telematic University Uninettuno, Corso Vittorio Emanuele II, 39, 00186 Rome, Italy; 3 Near East University, Operational Research Center in Healthcare, TRNC Mersin 10, Nicosia, 99138 Turkey; 4 Department of Computer Science and Mathematics, Lebanese American University, Beirut, Lebanon; 5 Department of Mathematics, Kurukshetra University, Kurukshetra, 136119 Haryana India; 6 Department of Quantitative Analysis, College of Business Administration, King Saud University, P.O. Box 71115, 11587 Riyadh, Saudi Arabia

**Keywords:** Engineering, Materials science, Mathematics and computing

## Abstract

This paper investigates the intricate energy distribution patterns emerging at an orthotropic piezothermoelastic half-space interface by considering the influence of a higher-order three-phase lags heat conduction law, accompanied by memory-dependent derivatives (referred to as HPS) within the underlying thermoelastic half-space (referred to as TS). This study explores the amplitude and energy ratios of reflected and transmitted waves. These waves span various incident types, including longitudinal, thermal, and transversal, as they propagate through the TS and interact at the interface. Upon encountering the interface, an intriguing dynamic unfolds: three waves experience reflection within the TS medium, while four waves undergo transmission into the HPS medium. A graphical representation effectively illustrates the impact of higher-order time differential parameters and memory to offer comprehensive insights. This visual representation reveals the nuanced fluctuations of energy ratios with the incidence angle. The model astutely captures diverse scenarios, showcasing its ability to interpret complex interface dynamics.

## Introduction

Many disciplines, including geophysics, earth-quake engineering, and seismology, have intensely interested in studying wave reflection and refraction phenomena. Studies of these phenomena are crucial for revealing the interior makeup of the Earth’s structure. They are significant when considering theoretical research and real-world applications in industries like mining and acoustics.

Fourier’s law of heat conduction provides a framework for the classical theory of thermoelasticity (CTE), developed by Duhamel. Fourier’s law produces the famous heat equation as the partial differential equation regulating heat transfer when coupled with the energy conservation law. There are two shortcomings in the CTE: first, the mechanical state of an elastic body does not affect the temperature, and second, the parabolic heat equation predicts an infinite propagation speed of heat. Biot^1^ proposed the model of coupled thermoelasticity, which stated that temperature changed independent of elastic variations and removed the first paradox of CTE. But the diffusion-type of heat conduction equation makes it difficult for the CTE and the Biot theory of thermoelasticity to describe the thermal signal velocity mechanism.

In 1967, to overcome this difficulty, Lord and Shulman (L–S)^2^ developed the generalized thermoelastic theory by incorporating one relaxation time into Fourier’s heat transfer law. Green and Lindsay^3^ developed the second generalized theory of thermoelasticity with two relaxation time parameters and included the temperature rate-dependent term in the heat equation. For the homogenous isotropic material, the three new thermoelastic models depending on the energy dissipation and thermal signal, were developed by Green and Naghdi^4–6^ and labeled as GN-I, GN-II, and GN-III. The linearized form of the GN-I model is the same as the CTE and displays the heat conduction paradox. The finite heat conduction speed without energy dissipation predicted by the GN-II model makes it the most controversial of the three. The GN-III model includes the preceding two models as exceptional cases. In GN-III model, a second sound may arise, but only when there is no dissipation, i.e., when the hyperbolic heat equation.

Tzou^7^ proposed the dual-phase-lag (DPL) heat conduction model by introducing two phase lag times $\tau_{q}$ (for heat flow) and $\tau_{T}$ (for temperature gradient) in the heat conduction equation. Microscopically, phonon-electron interaction in metallic films and phonon scattering in dielectric films, insulators, and semiconductors control heat transfer. Classical theories made from the macroscopic point of view, like heat diffusion based on Fourier’s law, are unlikely to be helpful at the microscale since they depict the macroscopic average behavior of numerous grains. Finite periods, from femtoseconds to seconds or even longer, are needed to complete the microstructural interactions. The lagged response describes the temperature gradient and the heat flow vector, which appear at various points in the heat transfer process. Roy Choudhuri^8^ extended the DPL model and presented the three-phase-lag (TPL) model by introducing the new phase lag time $\tau_{v}$ (for thermal displacement gradient).

In 2011, Wang and Li^9^ discovered the novel concept of memory-dependent derivatives (MDD) as a substitution of fractional order derivative, in which, using kernel function, the fractional derivative developed by Caputo and Mainardi^10^ was transformed into an integral form of derivative, and it can be written mathematically as

1 $$ D_{\tau } f(t) = \frac{1}{\tau }\int_{t - \tau }^{t} {\kappa \left( {t - \zeta } \right)f^{\prime}(\zeta )d\zeta .} $$

Here, the kernel function $\kappa (t - \zeta )$ and the time-delay parameter $\tau > 0$ can be chosen freely depending on the nature of the problem. From a physical perspective^11^, we usually assume that $0 < \kappa (t - \zeta ) \le 1$ and $\tau$ should be less than the upper limit set by the kernel function to make sure that the solution is unique and exists such that

2 $$ \kappa (t - \zeta ) = 1 - \frac{2\beta }{\tau }(t - \zeta ) + \frac{{\alpha^{2} }}{{\tau^{2} }}(t - \zeta )^{2} = \left\{ {\begin{array}{ll} 1 & {if\;\alpha = \beta = 0} \\ {1 - {{(t - \zeta )} \mathord{\left/ {\vphantom {{(t - \zeta )} \tau }} \right. \kern-0pt} \tau }} & {if\;\alpha = 0,\;\beta = {1 \mathord{\left/ {\vphantom {1 2}} \right. \kern-0pt} 2}} \\ {\left( {1 - {{(t - \zeta )} \mathord{\left/ {\vphantom {{(t - \zeta )} \tau }} \right. \kern-0pt} \tau }} \right)^{2} } & {if\;\alpha = \beta = 1} \\ \end{array} } \right.. $$

Also, Wang and Li^12,13^ proved that this concept is preferable to fractional calculus to display the instantaneous rate of change depending on the past state (memory effect). So many examples, like weather population models, forecasts, etc., need data from the recent past. This is only possible with the concept of MDD because fractional derivatives fail if the lower terminal value is much less than the upper terminal value in the definition of fractional order derivatives.

In the last few decades, piezo thermoelectric materials have gained interest in energy harvesting structures, actuators, transducers, dynamic sensing, surface acoustic wave, intelligent networks, mechanical systems, etc. Both experimental and theoretical studies on wave propagation in piezothermoelastic materials are active research subjects for researchers, scientists, and engineers. Mindlin^14^ was the first person who developed the piezothermoelectricity theory and its governing equation. Later, Nowacki^15^ and Chandrasekharaiah^16^ extended the physical law of piezothermoelectricity. Recently, Gupta and his team^17–23^ studied the various reflection and deformation problems at the interface of piezothermoelastic medium under different piezothermoelasticity theories. Several researchers, Barak et al.^24^ and Kumar et al.^25–27^, verified the energy balance law of incident, reflected and transmitted waves at the interface of various media. Li and his research team^28–31^ delved into the diverse challenges surrounding the thermo-electromechanical behavior of intricate piezoelectric smart nanocomposite structures by employing the size-dependent piezoelectric thermoelasticity theory and using the Laplace transformation technique.

To better understand the high-order consequences of thermal lagging, Chiriţă^32^ studied resonance phenomena under high-frequency excitations about micro or nanoscale heat transport models. This issue is significant when the model being considered has to account for interactions between various energy carriers as well as the impacts of the microstructural interactions that play a role in the quick and transitory transport of heat transient. Recently, Abouelregal and his team^33–36^ worked on several problems about the higher-order time differential of heat conduction equation by expanding Fourier’s law with Taylor’s series expansion. They successfully developed the idea of higher order time differential on various generalized thermoelastic theories such as L-S, GN-II, GN-III, DPL, and TPL under the presence and absence of MDD.

In this current manuscript, the energy ratios of various reflected/transmitted waves for incidence *P*, *T,* or *SV* at the interface $x_{3} = 0$ of orthotropic piezothermoelastic half-space in the context of a triple-phase lag heat conduction law with higher order MDD underlying a thermoelastic half-space are investigated. The impact of higher-order MDD on the various energy ratios is analyzed and depicted graphically.

## Basic equations

The constitutive relations for a homogenous, anisotropic piezothermoelastic solid under three-phase lag heat transfer law with higher-order MDD in the absence of free charge density, body forces, and shear forces are given by Abouelregal et al.^36^ and Barak and Gupta^37^, as

3 $$ \sigma_{ij} = c_{ijro} \;e_{ro} - \eta_{ijr} \;E_{r} - \beta_{ij} \;T, $$

4 $$ \sigma_{ij} ,_{j} = \rho \;\ddot{u}_{i} , $$

5 $$ E_{i} = - \;\phi ,_{i} , $$

6 $$ D_{i} = \varepsilon_{ij} E_{j} + \eta_{ijr} \;e_{jr} + p_{i} \;T, $$

7 $$ D_{i} ,_{i} = 0, $$

8 $$ K_{ij} \frac{\partial }{\partial t}\left( {1 + \sum\limits_{m = 1}^{p} {\frac{{\tau_{T}^{m} }}{m!}D_{{\tau_{T} }}^{m} } } \right)T,_{ij} + K_{ij}^{*} \left( {1 + \sum\limits_{m = 1}^{l} {\frac{{\tau_{v}^{m} }}{m!}D_{{\tau_{v} }}^{m} } } \right)T,_{ij} = \left( {1 + \sum\limits_{m = 1}^{n} {\frac{{\tau_{q}^{m} }}{m!}D_{{\tau_{q} }}^{m} } } \right)\left( {\rho C_{E} \ddot{T} + T_{0} \left( {\beta_{ij} \ddot{u}_{i} ,_{j} - p_{i} \ddot{\phi },_{i} } \right)} \right) $$

the higher orders $p,\,l,\,n\,\, \in \,{\rm N}$ and $i,\,j,\,r,\,o = 1,2,3$. Chiriţă et al.^38^ show that $n \ge 5$ or $p \ge 5$ leads to an unstable system, and therefore they cannot accurately describe an actual situation. Tzou^39^ presented a fascinating concept concerning this heat equation when $n = p$ or when $n = p - 1$ connecting the progressive exchange between the diffusive and wave behaviors.

The governing equations for a thermoelastic solid without energy dissipation and body forces are given by Green and Naghdi^6^ as:

9 $$ \left( {\lambda^{e} + \mu^{e} } \right)\nabla \left( {\nabla \cdot \vec{u}^{e} } \right) + \mu^{e} \nabla^{2} \vec{u}^{e} - \beta^{e} \nabla T^{e} = \rho^{e} \frac{{\partial^{2} \vec{u}^{e} }}{{\partial t^{2} }}, $$

10 $$ K_{e}^{*} \nabla^{2} T^{e} = \rho^{e} C_{E}^{e} \frac{{\partial^{2} T^{e} }}{{\partial t^{2} }} + \beta^{e} T_{0}^{e} \frac{{\partial^{2} }}{{\partial t^{2} }}\left( {\nabla \cdot \vec{u}^{e} } \right), $$

11 $$ \sigma_{ij}^{e} = \lambda^{e} u_{r,r} \delta_{ij} + \mu^{e} \left( {u_{i}^{e} ,_{j} + u_{j}^{e} ,_{i} } \right) - \beta^{e} T^{e} \delta_{ij} \;\;\;\left( {i,\,j,\,r = 1,2,3} \right)\,. $$

### Limiting cases

This work shows that the higher-order MDD piezothermoelastic proposed model is an extension of numerous generalized models, with or without the memory effect. The three-phase lag heat transfer law with higher order memory-dependent derivative Eq. (7) in the limiting case by setting.

**Case 1:** $\tau_{q} = \tau_{T} = \tau_{v} = 0$, and $K_{ij}^{*} = 0$ corresponds to Biot^1^ model.

**Case 2:** $n = 1$, $\tau_{q} > 0$, $\tau_{v} = \tau_{T} = 0$, $K_{ij}^{*} = 0$, $\kappa \left( {t - \zeta } \right) = 1$, and $D_{\tau } \to \frac{\partial }{\partial t}$ corresponds to Lord and Shulman’s^2^ model.

**Case 3:** $n \ge 1$, $\tau_{q} > 0$, $\tau_{v} = \tau_{T} = 0$, and $K_{ij}^{*} = 0$, transforms into the higher order MDD heat equation with one delay time $\tau_{q}$ developed by Abouelregal et al.^35^.

**Case 4:** $\tau_{q} = \tau_{T} = \tau_{v} = 0$, $K_{ij} = 0$, $\kappa \left( {t - \zeta } \right) = 1$, and $D_{\tau } \to \frac{\partial }{\partial t}$ corresponds to Green and Naghdi type-II model.

**Case 5:** $\tau_{q} = \tau_{T} = \tau_{v} = 0$, $\kappa \left( {t - \zeta } \right) = 1$, and $D_{\tau } \to \frac{\partial }{\partial t}$ corresponds to Green and Naghdi type-III model.

**Case 6:** $p = 1$, $n = 2$, $K_{ij}^{*} = \tau_{v} = 0$, $\kappa \left( {t - \zeta } \right) = 1$, and $D_{\tau } \to \frac{\partial }{\partial t}$ corresponds to Tzou^7^ model.

**Case 7:** $p = l = 1$, $n = 2$, $K_{ij}^{*} = \tau_{v} = 0$ corresponds to Ezzat et al.^40^ model.

**Case 8:** $n,\,p \ge 1$, $K_{ij}^{*} = \tau_{v} = 0$ transforms into the dual phase-lags heat conduction equation with higher order MDD developed by Abouelregal^41^.

**Case 9:** $p = l = 1$, $n = 2$, $\kappa \left( {t - \zeta } \right) = 1$, and $D_{\tau } \to \frac{\partial }{\partial t}$ corresponds to Roy Choudhuri^8^ model.

**Case 10:** $p = l = 1$, and $n = 2$, corresponds to Ghosh et al.^42^ model.

**Case 11:** $n,p,l \ge 1$, $\kappa \left( {t - \zeta } \right) = 1$, and $D_{\tau } \to \frac{\partial }{\partial t}$ change into the higher-order time derivative three-phase lag heat equation without MDD proposed by Abouelregal^33^.

### Nomenclature

**Table:** 

$c_{ijro} =$ elastic stiffness tensor	$\beta_{ij} =$ thermal moduli tensors
$\eta_{ijr} ,\,\varepsilon_{ij} =$ piezothermal moduli tensors	$E_{i} =$ electric field density
$\rho =$ density	$D_{i} =$ electric displacement
$e_{ij} =$ component of strain	$\lambda ,\,\mu =$ Lame’s constant
$\omega \,\,\, =$ circular frequency	$C_{E} =$ specific heat at constant strain
$K_{ij} =$ components of thermal conductivity	$K_{ij}^{*} =$ heat conduction tensor
$\tau_{q} =$ phase lag of heat flux	$K_{e}^{*} =$ material constant
$\tau_{v} =$ phase lag of thermal disp. gradient	$p_{i} =$ pyroelectric constants
$\tau_{T} =$ phase lag of the temperature gradient	$\phi =$ electrical potential
$\sigma_{ij} =$ components of the stress	$T =$ thermal temperature
$T_{0} =$ reference temperature	

for clarity, engineering notations are employed, and the terms partial derivative with respect to time or the corresponding Cartesian coordinate are denoted by a superimposed dot “.” or a subscript followed by a comma “,”, respectively. A superscript “*e*” denotes thermoelastic half-space parameters.

## Formulation of the problem

**Figure 1:**
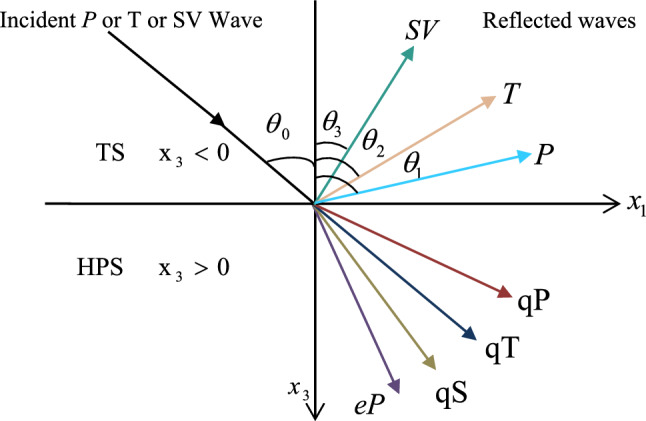
Refraction and reflection of plane wave in TS and HPS.

As illustrated in Fig. 1, the orthotropic piezothermoelastic half-space under the reference of a triple-phase lag theory of higher order memory-dependent derivatives (HPS) ($x_{3} > 0$) underlying a thermoelastic half-space (TS) ($x_{3} < 0$) are welded together. The thermoelastic plane wave propagates in the $x_{1} x_{3} -$ plane, and the displacement vector for HPS and TS is represented by $\vec{u} = (u_{1} ,0,u_{3} )$ and $\vec{u}^{e} = (u_{1}^{e} ,0,u_{3}^{e} )$ respectively.

Following Slaughter^43^, the governing equations for two-dimensional HPS medium are determined from Eqs. (3)–(8) as

12 $$ c_{11} u_{1} ,_{11} + c_{55} u_{1} ,_{33} + \left( {c_{13} + c_{55} } \right)u_{3} ,_{13} + \left( {\eta_{31} + \eta_{15} } \right)\phi ,_{13} - \beta_{1} T,_{1} = \rho \ddot{u}_{1} , $$

13 $$ \left( {c_{13} + c_{55} } \right)u_{1} ,_{13} c_{55} u_{3} ,_{11} + c_{33} u_{3} ,_{33} + \eta_{15} \phi ,_{11} + \eta_{33} \phi ,_{33} - \beta_{3} T,_{3} = \rho \ddot{u}_{3} , $$

14 $$ \frac{\partial }{\partial t}\left( {1 + \sum\limits_{m = 1}^{p} {\frac{{\tau_{T}^{m} }}{m!}D_{{\tau_{T} }}^{m} } } \right)\left( {K_{1} T,_{11} + K_{3} T,_{33} } \right) + \left( {1 + \sum\limits_{m = 1}^{l} {\frac{{\tau_{v}^{m} }}{m!}D_{{\tau_{v} }}^{m} } } \right)\left( {K_{1}^{*} T,_{11} + K_{3}^{*} T,_{33} } \right) = \left( {1 + \sum\limits_{m = 1}^{n} {\frac{{\tau_{q}^{m} }}{m!}D_{{\tau_{q} }}^{m} } } \right)\left( {\rho C_{E} \ddot{T} + T_{0} \left( {\beta_{1} \ddot{u}_{1} ,_{1} + \beta_{3} \ddot{u}_{3} ,_{3} - p_{3} \ddot{\phi },_{3} } \right)} \right), $$

15 $$ \left( {\eta_{15} + \eta_{31} } \right)u,_{13} + \eta_{15} u_{3} ,_{13} + \eta_{33} u_{3} ,_{33} - \varepsilon_{11} \phi ,_{11} - \varepsilon_{33} \phi ,_{33} + p_{3} T,_{3} = 0, $$

where $K_{ij} = K_{i} \delta_{ij}$, $K_{ij} = K_{i}^{*} \delta_{ij}$, $\beta_{ij} = \beta_{i} \delta_{ij} ,$ and $i$ is not summed.

For convenience, the dimensionless quantities are taken as

16 $$ \left( {x_{1}^{\prime } ,\,x_{3}^{\prime } ,} \right) = \frac{{\omega_{\,1} }}{{c_{1} }}\;\left( {x_{1} ,\,x_{3} } \right),\;\left( {u_{1}^{\prime } ,\,u_{3}^{\prime } ,\,u_{1}^{{e^{\prime}}} ,\,u_{3}^{{e^{\prime}}} } \right) = \frac{{\omega_{\,1} }}{{c_{1} }}\,\left( {\,u_{1} ,\,u_{3} ,\,u_{1}^{e} ,u_{3}^{e} } \right),t^{\prime} = \omega_{1} t,\;\left( {\sigma_{ij}^{\prime } ,\sigma_{ij}^{{e^{\prime}}} } \right) = \frac{1}{{\beta_{1} \,T_{0} }}\;\left( {\sigma_{ij} ,\sigma_{ij}^{e} } \right),\;\left( {\,\tau_{T}^{\prime } ,\,\tau_{q}^{\prime } ,\;\tau_{v}^{\prime } } \right) = \omega_{1} \,\left( {\,\,\tau_{T} ,\,\tau_{q} ,\tau_{v} } \right),\;\phi^{\prime} = \frac{{\omega_{\,1} \,\eta_{31} }}{{c_{1} \,\beta_{1} T_{0} }}\phi ,\;T^{\prime} = \frac{{\beta_{1} }}{{\rho \,c_{1}^{2} }}\,T\;{\text{where}}\;c_{1} = \sqrt {\frac{{c_{11} }}{\rho }} ,\;\omega_{\,1} = \frac{{\rho \,C_{E} \;c_{1}^{2} }}{{K_{1} }} $$

Using the Eq. (16) in the set of Eqs. (10)–(11) and (12)–(15) with the removal of primes (′) takes the following form

17 $$ \left( {\frac{{\partial^{2} }}{{\partial x_{1}^{2} }} + d_{11} \frac{{\partial^{2} }}{{\partial x_{3}^{2} }} - \frac{{\partial^{2} }}{{\partial t^{2} }}} \right)u_{1} + d_{12} \frac{{\partial^{2} u_{3} }}{{\partial x_{1} \partial x_{3} }} + d_{13} \frac{{\partial^{2} \phi }}{{\partial x_{1} \partial x_{3} }} - \frac{\partial T}{{\partial x_{1} }} = 0, $$

18 $$ \frac{{\partial^{2} u_{1} }}{{\partial x_{1} \partial x_{3} }} + \left( {d_{21} \frac{{\partial^{2} }}{{\partial x_{1}^{2} }} + d_{22} \frac{{\partial^{2} }}{{\partial x_{3}^{2} }} - d_{23} \frac{{\partial^{2} }}{{\partial t^{2} }}} \right)u_{3} + \left( {d_{24} \frac{{\partial^{2} }}{{\partial x_{1}^{2} }} + d_{25} \frac{{\partial^{2} }}{{\partial x_{3}^{2} }}} \right)\phi - d_{26} \frac{\partial T}{{\partial x_{3} }} = 0, $$

19 $$ \left( {1 + \sum\limits_{m = 1}^{l} {\frac{{\tau_{v}^{m} }}{m!}D_{{\tau_{v} }}^{m} } } \right)\left( {d_{31} \frac{{\partial^{2} }}{{\partial x_{1}^{2} }} + d_{32} \frac{{\partial^{2} }}{{\partial x_{3}^{2} }}} \right)T + \frac{\partial }{\partial t}\left( {1 + \sum\limits_{m = 1}^{p} {\frac{{\tau_{T}^{m} }}{m!}D_{{\tau_{T} }}^{m} } } \right)\left( {d_{33} \frac{{\partial^{2} }}{{\partial x_{1}^{2} }} + d_{34} \frac{{\partial^{2} }}{{\partial x_{3}^{2} }}} \right)T = \left( {1 + \sum\limits_{m = 1}^{n} {\frac{{\tau_{q}^{m} }}{m!}D_{{\tau_{q} }}^{m} } } \right)\left( {\frac{{\partial^{2} \ddot{u}_{1} }}{{\partial x_{1}^{2} }} + d_{35} \frac{{\partial^{2} \ddot{u}_{3} }}{{\partial x_{3}^{2} }} - d_{36} \frac{{\partial \ddot{\phi }}}{{\partial x_{3} }} + d_{37} \ddot{T}} \right) $$

20 $$ \frac{{\partial^{2} u_{1} }}{{\partial x_{1} \partial x_{3} }} + \left( {d_{41} \frac{{\partial^{2} u_{3} }}{{\partial x_{1}^{2} }} + d_{42} \frac{{\partial^{2} u_{3} }}{{\partial x_{3}^{2} }}} \right) - \left( {d_{43} \frac{{\partial^{2} \phi }}{{\partial x_{1}^{2} }} + d_{44} \frac{{\partial^{2} \phi }}{{\partial x_{3}^{2} }}} \right) + d_{45} \frac{\partial T}{{\partial x_{3} }} = 0, $$

21 $$ \left( {\frac{{\alpha_{1}^{e2} - \alpha_{2}^{e2} }}{{c_{1}^{2} }}} \right)\left( {\frac{{\partial^{2} u_{1}^{e} }}{{\partial x_{1}^{2} }} + \frac{{\partial^{2} u_{3}^{e} }}{{\partial x_{3} \,\partial x_{1} }}} \right) + \left( {\frac{{\alpha_{2}^{e2} }}{{c_{1}^{2} }}} \right)\left( {\frac{{\partial^{2} u_{1}^{e} }}{{\partial x_{1}^{2} }} + \frac{{\partial^{2} u_{1}^{e} }}{{\partial x_{3}^{2} }}} \right) - \gamma \frac{{\partial T^{e} }}{{\partial x_{1} }} = \frac{{\partial^{2} u_{1}^{e} }}{{\partial t^{2} }}, $$

22 $$ \left( {\frac{{\alpha_{1}^{e2} - \alpha_{2}^{e2} }}{{c_{1}^{2} }}} \right)\left( {\frac{{\partial^{2} u_{1}^{e} }}{{\partial x_{3} \,\partial x_{1} }} + \frac{{\partial^{2} u_{3}^{e} }}{{\partial x_{3}^{2} }}} \right) + \left( {\frac{{\alpha_{2}^{e2} }}{{c_{1}^{2} }}} \right)\left( {\frac{{\partial^{2} u_{3}^{e} }}{{\partial x_{1}^{2} }} + \frac{{\partial^{2} u_{3}^{e} }}{{\partial x_{3}^{2} }}} \right) - \gamma \frac{{\partial T^{e} }}{{\partial x_{3} }} = \frac{{\partial^{2} u_{3}^{e} }}{{\partial t^{2} }}, $$

23 $$ \left( {\frac{\partial }{{\partial x_{1} }} + \frac{\partial }{{\partial x_{3} }}} \right)\ddot{u}^{e} - d_{51} \left( {\frac{\partial }{{\partial x_{1}^{2} }} + \frac{\partial }{{\partial x_{3}^{2} }}} \right)T^{e} + d_{52} \frac{{\partial^{2} T^{e} }}{{\partial t^{2} }} = 0, $$

where $$d_{11} = \frac{{c_{55} }}{{c_{11} }}$$, $$d_{12} = \frac{{c_{13} + c_{55} }}{{c_{11} }}$$, $$d_{13} = \frac{{\eta_{31} + \eta_{15} }}{{c_{11} \eta_{31} }}\beta_{1} T_{0}$$, $$d_{21} = \frac{{c_{55} }}{{c_{55} + c_{13} }}$$, $$d_{22} = \frac{{c_{33} }}{{c_{55} + c_{13} }}$$ $$d_{23} = \frac{{c_{11} }}{{c_{55} + c_{13} }}$$, $$d_{24} = \frac{{\eta_{15} \beta_{1} T_{0} }}{{\eta_{31} (c_{55} + c_{13} )}}$$, $$d_{25} = \frac{{\eta_{33} \beta_{1} T_{0} }}{{\eta_{31} (c_{55} + c_{13} )}}$$, $$d_{26} = \frac{{c_{11} \beta_{3} }}{{(c_{55} + c_{13} )\beta_{1} }}$$, $$d_{31} = \frac{{K_{1}^{*} \rho }}{{T_{0} \beta_{1}^{2} }}$$ $$d_{32} = \frac{{K_{3}^{*} \rho }}{{T_{0} \beta_{1}^{2} }}$$, $$d_{33} = \frac{{K_{1} \omega_{1} \rho }}{{T_{0} \beta_{1}^{2} }}$$, $$d_{34} = \frac{{K_{3} \omega_{1} \rho }}{{T_{0} \beta_{1}^{2} }}$$, $$d_{35} = \frac{{\beta_{3} }}{{\beta_{1} }}$$, $$d_{36} = \frac{{p_{3} T_{0} }}{{\eta_{31} }}$$, $$d_{37} = \frac{{c_{11} \rho C_{E} }}{{T_{0} \beta_{1}^{2} }}$$ $$d_{41} = \frac{{\eta_{15} }}{{(\eta_{15} + \eta_{31} )}}$$, $$d_{42} = \frac{{\eta_{33} }}{{(\eta_{15} + \eta_{31} )}}$$, $$d_{43} = \frac{{\varepsilon_{11} \beta_{1} T_{0} }}{{\eta_{31} (\eta_{15} + \eta_{31} )}}$$, $$d_{44} = \frac{{\varepsilon_{33} \beta_{1} T_{0} }}{{\eta_{31} (\eta_{15} + \eta_{31} )}}$$, $$\alpha_{2}^{e} = \sqrt {\frac{{\mu^{e} }}{{\rho^{e} }}}$$$$d_{45} = \frac{{c_{11} \tau_{3} }}{{\beta_{1} (\eta_{15} + \eta_{31} )}}$$, $$d_{51} = \frac{{K_{e}^{*} \rho }}{{\beta_{1} \beta^{e} T_{0}^{e} }}$$, $$d_{52} = \frac{{\rho^{e} \rho C_{E}^{e} c_{1}^{2} }}{{\beta_{1} \beta^{e} T_{0}^{e} }}$$, $$\alpha_{1}^{e} = \sqrt {\frac{{\lambda^{e} + 2\mu^{e} }}{{\rho^{e} }}}$$, $$\gamma = \frac{{\rho \beta^{e} }}{{\rho^{e} \beta_{1} }}$$.

## Method (wave propagation analysis)

Taking plane wave form solution for HPS medium as

24 $$ \left( {u_{1} ,\,u_{3} ,\,\phi ,\,\,T} \right)\left( {x_{1} ,x_{3} ,t} \right)\, = \left( {\,H,\,M,\,N,\,U} \right)\,\exp \left[ {\iota \omega \left( { - \frac{{x_{1} }}{c} - q\;x_{3} + t} \right)} \right], $$

where, $H,\,M,\,N,\,U$ are the amplitude vectors, $q$ and $c$ are the slowness parameter, and the apparent phase velocity, respectively.

Inserting Eq. (24) in a set of Eqs. (17)–(20) yields a homogenous system

25 $$ \Omega R = 0 $$

26 $$ \Omega = \left[ {\begin{array}{*{20}c} {l_{11} + q^{2} l_{12} } & {ql_{13} } & {ql_{14} } & { - l_{15} } \\ {qc} & {l_{21} + q^{2} l_{22} } & {d_{24} + q^{2} l_{23} } & { - ql_{24} } \\ c & {ql_{31} } & { - ql_{32} } & {l_{33} + q^{2} l_{34} } \\ {cq} & {d_{41} + q^{2} l_{41} } & { - d_{43} - q^{2} l_{42} } & {ql_{43} } \\ \end{array} } \right],\;R = \left[ \begin{gathered} H \hfill \\ M \hfill \\ N \hfill \\ U \hfill \\ \end{gathered} \right], $$

where,

$$l_{11} = c - c^{3}$$, $$l_{12} = d_{11} c^{3}$$, $$l_{13} = d_{12} c^{2}$$, $$l_{14} = d_{13} c^{2}$$, $$l_{15} = \frac{{\iota c^{2} }}{\omega }$$, $$l_{21} = d_{21} - d_{23} c^{2}$$, $$l_{22} = d_{22} c^{2}$$, $$l_{23} = d_{25} c^{2}$$, $$l_{24} = \frac{{\iota d_{26} \,c^{2} }}{\omega }$$, $$l_{31} = d_{25} c^{2}$$, $$l_{32} = d_{36} c^{2}$$, $$l_{33} = \frac{{A_{1} d_{31} }}{{\iota \omega^{2} }} + A_{2} d_{33} - \frac{{d_{37} c^{2} }}{\iota \omega }$$, $$l_{34} = \left( {A_{2} d_{34} - \frac{{\iota A_{1} d_{32} }}{\omega }} \right)c^{2}$$, $$l_{41} = d_{42} c^{2}$$, $$l_{42} = d_{44} c^{2}$$, $$l_{43} = \frac{{\iota c^{2} d_{45} }}{\omega }$$, $$A_{1} = \frac{{1 + \Gamma_{1} }}{{1 + \Gamma_{2} }},$$ $$A_{2} = \frac{{1 + \Gamma_{3} }}{{1 + \Gamma_{2} }}$$, $$\Gamma_{1} = \sum\limits_{m = 1}^{p} {\frac{{\tau_{T}^{m} }}{m!}b^{m - 1} \Gamma \left( {\tau_{T} ,b} \right)}$$, $$\Gamma_{2} = \sum\limits_{m = 1}^{n} {\frac{{\tau_{q}^{m} }}{m!}b^{m - 1} \Gamma \left( {\tau_{q} ,b} \right)}$$, $$\Gamma_{3} = \sum\limits_{m = 1}^{l} {\frac{{\tau_{v}^{m} }}{m!}b^{m - 1} \Gamma \left( {\tau_{v} ,b} \right)}$$, $$b = \iota \omega$$

$$ \Gamma \left( {\tau_{i} ,b} \right) = \frac{{\left( { - b^{2} \tau_{i}^{2} \left( {\alpha^{2} - 2\beta + 1} \right) + 2b\tau_{i} \left( {\alpha^{2} - \beta } \right) + 2\alpha^{2} } \right)\exp \left( { - b\tau_{i} } \right) + b^{2} \tau_{i}^{2} - 2b\beta \tau_{i} + 2\alpha^{2} }}{{b^{2} \tau_{i}^{3} }},\,\tau_{i} = \tau_{v} ,\,\tau_{T} ,\,\tau_{q} . $$

The coefficient matrix’s $\Omega$ determinant must be zero for the non-trivial solution, leading to a characteristic equation.

27 $$ G_{11} \,q^{8} + G_{12} \,q^{6} + G_{13} \,q^{4} + G_{14} \,q^{2} + G_{15} = 0\,. $$

When Eq. (27) is solved using the MATLAB software, the roots are found to be organized in magnitude-wise decreasing order and are denoted as follows for convenience: $q_{i} \,\,\left( {i = 1 - 4} \right)$ represents the roots of this equation with positive imaginary parts and $q_{i} \,\,\left( {i = 5 - 8} \right)$ represents those with negative imaginary parts. The eigenvalue $q_{4}$ corresponds to the component of the electric potential $\,(\,eP\,)$ mode of wave propagation and $q_{i} \,\,\left( {i = 1 - 3} \right)$ corresponds to the quasi $P\,\,(\,qP\,)$, quasi $T\,\,(\,qT\,)$, and quasi $S\,\,(\,qS\,)$ propagating modes, respectively. The expression $G_{1i} \,\,\left( {i = 1 - 5} \right)$ is provided in the Appendix.

The eigenvectors $H_{i}$, $M_{i}$, $N_{i}$, and $U_{i}$ corresponding to each $q_{i} \,\,\left( {i = 1 - 8} \right)$ can be written as

28 $$ W_{i} = \frac{{cf\,\left( {\Omega_{42} } \right)_{{q_{i} }} }}{{cf\,\left( {\Omega_{41} } \right)_{{q_{i} }} }},\;\Phi_{i} = \,\frac{{cf\,\left( {\Omega_{43} } \right)_{{q_{i} }} }}{{cf\,\left( {\Omega_{41} } \right)_{{q_{i} }} }},\;\Theta_{i} = \frac{{cf\,\left( {\Omega_{44} } \right)_{{q_{i} }} }}{{cf\,\left( {\Omega_{41} } \right)_{{q_{i} }} }}, $$

where

29 $$ W_{i} = \frac{{M_{i} }}{{H_{i} }},\;\Phi_{i} = \frac{{N_{i} }}{{H_{i} }},\;\Theta_{i} = \frac{{U_{i} }}{{H_{i} }}, $$

and $cf\,\left( {\Omega_{ij} } \right)_{{q_{i} }}$ denote the cofactor $G_{ij}$ corresponding to the eigenvalue $q_{i}$. The corresponding amplitudes $\left( {H_{i} ,\,M_{i} ,\,\,N_{i} {\text{ and}}\,\,U_{i} } \right)$ decrease when the thermoelastic plane waves go through the HPS medium, depending on the frequency of these waves.

Following Achenbach^44^, the displacement components of the TS medium can be expressed in terms of the potential function $\phi^{e}$ and $\psi^{e}$ by the relation

30 $$ u_{1}^{e} = \frac{{\partial \phi^{e} }}{{\partial x_{1} }} - \frac{{\partial \psi^{e} }}{{\partial x_{3} }},\;u_{3}^{e} = \frac{{\partial \phi^{e} }}{{\partial x_{3} }} + \frac{{\partial \psi^{e} }}{{\partial x_{1} }}. $$

Using Eq. (30) in Eqs. (21)–(23), we get

31 $$ \alpha_{1}^{*2} \nabla^{2} \phi^{e} - \ddot{\phi }^{e} - \gamma T^{e} = 0, $$

32 $$ \alpha_{2}^{*2} \nabla^{2} \psi^{e} - \ddot{\psi }^{e} = 0, $$

33 $$ a_{1}^{2} \nabla^{2} T^{e} - \ddot{T}^{e} - a_{2}^{2} \nabla^{2} \ddot{\phi }^{e} = 0, $$

where, $$\alpha_{1}^{*} = \frac{{\alpha_{1}^{e} }}{{c_{1} }}$$, $$\alpha_{2}^{*} = \frac{{\alpha_{2}^{e} }}{{c_{1} }}$$, $$a_{1}^{2} = \frac{{K_{c}^{*} }}{{\rho^{e} c_{1}^{2} C^{e} }}$$, $$a_{2}^{2} = \frac{{\beta^{e} \beta_{1} T_{0}^{e} }}{{\rho \rho^{e} c_{1}^{2} C_{E}^{e} }}$$.

For thermoelastic plane harmonic wave propagating in TS medium establishing an angle $\theta_{0}$ with the $x_{3} -$ axis (see Fig. 1), we can take

34 $$ \left( {\phi^{e} ,T^{e} ,\psi^{e} } \right) = \left( {A_{{\phi^{e} }} ,A_{{T^{e} }} ,A_{{\psi^{e} }} } \right)\exp \left[ {\iota k\left( { - x_{1} \sin \theta_{0} - x_{3} \cos \theta_{0} } \right) + \iota \omega t} \right] $$

where $k( = {\omega \mathord{\left/ {\vphantom {\omega c}} \right. \kern-0pt} c})$ is the complex wavenumber, $A_{{\phi^{e} }}$, $A_{{T^{e} }}$, $A_{{\psi^{e} }}$ are the wave amplitudes constants.

Using Eq. (34) in Eqs. (31)–(33), we get

35 $$ r_{{e_{1,2} }}^{2} = \frac{{S_{1} \pm \sqrt {S_{1}^{2} - 4S_{2} } }}{2},\;r_{{e_{3} }}^{2} = \alpha_{2}^{*2} , $$

where $$S_{1} = a_{1}^{2} + \alpha_{1}^{*2} + a_{2}^{2} \gamma$$, $$S_{2} = \alpha_{1}^{*2} a_{1}^{2} .$$

Here $r_{{e_{1} }}^{2}$, $r_{{e_{2} }}^{2}$ corresponds to positive and negative signs, respectively. These roots indicate that two coupled longitudinal waves exist, namely, an elastic wave (*P*-wave) and a thermal wave (*T*-wave). $r_{{e_{3} }}^{2}$ corresponds to transversal *SV-* wave.

## Refraction and reflection coefficients

### Amplitude ratios

Assuming that train of a thermoelastic plane wave (*P* or *SV* or *T*) striking at the interface via the TS medium form an angle $\theta_{0}$ with the $x_{3} -$ axis, causing three waves to be reflected in the TS medium and four waves to be transmitted in the HPS medium. The expression for the stress, electric displacement, mechanical displacements, electric potential, and temperature assumed in an HPS medium becomes

36 $$ \left( {\sigma_{33} ,\,\sigma_{31} ,\,D_{3} } \right) = \,\iota \,\omega \,\sum\limits_{i = 1}^{4} {\left( {\Delta_{1i} ,\,\Delta_{2i} ,\,\Delta_{7i} } \right)} \,H_{i} \,\exp \left[ {\iota \,\omega \,\left( { - \frac{{x_{1} }}{c} - q\,x_{3} + t} \right)} \right], $$

37 $$ \left( {u_{1} ,\,u_{3} ,\,\phi ,\,T} \right) = \sum\limits_{i = 1}^{4} {\left( {1,\,W_{i} ,\,\Phi_{i} ,\,\Theta_{i} } \right)\,H_{i} \,\exp \left( {\iota \omega \left( { - \frac{{x_{1} }}{c} - q_{i} \,x_{3} + t} \right)} \right)} . $$

### Boundary conditions

The possible boundary conditions, *i.e.,* equality of distribution of normal stress, tangential stress, tangential displacement, and normal displacement, along with the isothermal, insulated boundaries, and vanishing of electric displacements across an interface, $x_{3} = 0$ are as follows:

38 $$ \sigma_{33} = \sigma_{33}^{e} ,\,\,\sigma_{31} = \sigma_{31}^{e} ,\,\,u_{1} = u_{1}^{e} ,\,\,u_{3} = u_{3}^{e} ,\,\,T = T^{e} ,\left[ {K_{3} \frac{\partial }{\partial t}\left( {1 + \sum\limits_{m = 1}^{p} {\frac{{\tau_{T}^{m} }}{m!}D_{{\tau_{T} }}^{m} } } \right) + K_{3}^{*} \left( {1 + \sum\limits_{m = 1}^{l} {\frac{{\tau_{v}^{m} }}{m!}D_{{\tau_{v} }}^{m} } } \right)} \right]\frac{\partial T}{{\partial x_{3} }} = K_{e}^{*} \frac{{\partial T^{e} }}{{\partial x_{3} }},\;D_{3} = 0. $$

The full structures of the wave field made up of the incident and reflected wave in the TS medium meet the boundary conditions, are

39 $$ \begin{gathered} \phi^{e} = A_{0}^{e} \exp \left\{ {ik_{1} \left( { - x_{1} \sin \theta_{0} - x_{3} \cos \theta_{0} } \right) + \iota \omega t} \right\} + A_{1}^{e} \exp \left\{ {ik_{1} \left( { - x_{1} \sin \theta_{1} + x_{3} \cos \theta_{1} } \right) + \iota \omega t} \right\} \\ + B_{0}^{e} \exp \left\{ {ik_{2} \left( { - x_{1} \sin \theta_{0} - x_{3} \cos \theta_{0} } \right) + \iota \omega t} \right\} + B_{1}^{e} \exp \left\{ {ik_{2} \left( { - x_{1} \sin \theta_{2} + x_{3} \cos \theta_{2} } \right) + \iota \omega t} \right\}, \\ \end{gathered} $$

40 $$ \begin{gathered} T^{e} = \zeta_{1} A_{0}^{e} \exp \left\{ {ik_{1} \left( { - x_{1} \sin \theta_{0} - x_{3} \cos \theta_{0} } \right) + \iota \omega t} \right\} + \zeta_{1} A_{1}^{e} \exp \left\{ {ik_{1} \left( { - x_{1} \sin \theta_{1} + x_{3} \cos \theta_{1} } \right) + \iota \omega t} \right\} \\ + \zeta_{2} B_{0}^{e} \exp \left\{ {ik_{2} \left( { - x_{1} \sin \theta_{0} - x_{3} \cos \theta_{0} } \right) + \iota \omega t} \right\} + \zeta_{2} B_{1}^{e} \exp \left\{ {ik_{2} \left( { - x_{1} \sin \theta_{2} + x_{3} \cos \theta_{2} } \right) + \iota \omega t} \right\}, \\ \end{gathered} $$

41 $$ \psi^{e} = D_{0}^{e} \exp \left\{ {ik_{3} \left( { - x_{1} \sin \theta_{0} - x_{3} \cos \theta_{0} } \right) + \iota \omega t} \right\} + D_{1}^{e} \exp \left\{ {ik_{3} \left( { - x_{1} \sin \theta_{3} + x_{3} \cos \theta_{3} } \right) + \iota \omega t} \right\}, $$

where $\zeta_{j} = \frac{{\omega^{2} }}{{r_{{e_{j} }}^{2} \gamma }}\left( {r_{{e_{j} }}^{2} - \alpha_{1}^{*2} } \right)$, $j = 1,\,2$ and $A_{0}^{e}$, $B_{0}^{e}$, $D_{0}^{e}$ ($A_{1}^{e}$, $B_{1}^{e}$, $D_{1}^{e}$) represent the coefficients of amplitudes of the incident (reflected) *P-*, *T-*, and *SV-* waves, respectively and

$B_{0}^{e} ,\,D_{0}^{e} = 0$, for incidence of *P-*wave.

$A_{0}^{e} ,\,D_{0}^{e} = 0$, for incidence of *T-*wave.

$A_{0}^{e} ,\,B_{0}^{e} = 0$, for incidence of *SV-*wave.

Snell’s law is given as

42 $$ \frac{{\sin \theta_{0} }}{{V_{0} }} = \frac{{\sin \theta_{1} }}{{r_{{e_{1} }} }} = \frac{{\sin \theta_{2} }}{{r_{{e_{2} }} }} = \frac{{\sin \theta_{3} }}{{r_{{e_{3} }} }}, $$

where,

43 $$ V_{0} = \left\{ {\begin{array}{*{20}c} {r_{{e_{1} }} \,{\text{for}}\,\,{\text{incident}}\,{\text{of}}\,\,P\, - {\text{wave}}} \\ {r_{{e_{2} }} \,{\text{for}}\,\,{\text{incident}}\,{\text{of}}\,\,T\, - {\text{wave}}} \\ {r_{{e_{3} }} \,{\text{for}}\,\,{\text{incident}}\,{\text{of}}\,\,SV\, - {\text{wave}}} \\ \end{array} } \right.. $$

Using the set of Eqs. (36)–(43), we obtain a system of non-homogenous equations that may be expressed as

44 $$ \Delta X = Q, $$

where

$$ \Delta = \left[ {\begin{array}{*{20}c} {\Delta_{11} } & {\Delta_{12} } & {\Delta_{13} } & {\Delta_{14} } & {\Delta_{15} } & {\Delta_{16} } & {\Delta_{17} } \\ {\Delta_{21} } & {\Delta_{22} } & {\Delta_{23} } & {\Delta_{24} } & {\Delta_{25} } & {\Delta_{26} } & {\Delta_{27} } \\ {\Delta_{31} } & {\Delta_{32} } & {\Delta_{33} } & {\Delta_{34} } & {\Delta_{35} } & {\Delta_{36} } & {\Delta_{37} } \\ {\Delta_{41} } & {\Delta_{42} } & {\Delta_{43} } & {\Delta_{44} } & {\Delta_{45} } & {\Delta_{46} } & {\Delta_{47} } \\ {\Delta_{51} } & {\Delta_{52} } & {\Delta_{53} } & {\Delta_{54} } & {\Delta_{55} } & {\Delta_{56} } & 0 \\ {\Delta_{61} } & {\Delta_{62} } & {\Delta_{63} } & {\Delta_{64} } & {\Delta_{65} } & {\Delta_{66} } & 0 \\ {\Delta_{71} } & {\Delta_{72} } & {\Delta_{73} } & {\Delta_{74} } & 0 & 0 & 0 \\ \end{array} } \right],\;X = \left[ {\begin{array}{*{20}c} {X_{1} } \\ {X_{2} } \\ {X_{3} } \\ {X_{4} } \\ {X_{5} } \\ {X_{6} } \\ {X_{7} } \\ \end{array} } \right],\;Q = \left[ {\begin{array}{*{20}c} {Q_{1} } \\ {Q_{2} } \\ {Q_{3} } \\ {Q_{4} } \\ {Q_{5} } \\ {Q_{6} } \\ 0 \\ \end{array} } \right]. $$

$$\Delta_{1i} = - \frac{{c_{13} }}{{\beta_{1} T_{0} c}} - \left( {\frac{{c_{33} W_{i} }}{{\beta_{1} T_{0} }} + \frac{{\eta_{33} }}{{\eta_{31} }}\Phi_{i} } \right)q_{i} - \frac{{c_{11} \beta_{3} }}{{\iota \omega \beta_{1}^{2} T_{0} }}\Theta_{i}$$, $$\Delta_{2i} = - \frac{{c_{55} W_{i} }}{{\beta_{1} T_{0} c}} - \frac{{c_{55} q_{i} }}{{\beta_{1} T_{0} }} - \frac{{\eta_{15} \Phi_{i} }}{{c\eta_{31} }}$$, $$\Delta_{3i} = 1$$, $$\Delta_{4i} = W_{i}$$, $$\Delta_{5i} = \Theta_{i}$$, $$\Delta_{6i} = - \left[ {K_{3}^{*} \left( {1 + \tau_{v} \Gamma \left( {\tau ,b} \right)} \right) + K_{3} \left( {1 + \tau_{T} \Gamma \left( {\tau ,b} \right)} \right)\iota \omega \,\omega_{1} } \right]\Theta_{i} q_{i}$$

$$ \Delta_{7i} = - \frac{{c_{11} \eta_{31} }}{{\beta_{1} T_{0} \eta_{33} c}} - \frac{{c_{11} q_{i} W_{i} }}{{\beta_{1} T_{0} }} + \frac{{c_{11} \varepsilon_{33} }}{{\eta_{33} \eta_{31} }}q_{i} \Phi_{i} + \frac{{p_{3} c_{11}^{2} }}{{\iota \omega \eta_{33} \beta_{1}^{2} T_{0} }}\Theta_{i} ,\;\;i = 1 - 4, $$

$$\Delta_{15} = \frac{{C_{T} \zeta_{1} + k_{1}^{2} \rho^{e} \left( {\alpha_{1}^{e2} - 2\alpha_{2}^{e2} \sin^{2} \theta_{1} } \right)}}{{\iota \omega \beta_{1} T_{0} }}$$, $$\Delta_{16} = \frac{{C_{T} \zeta_{2} + k_{2}^{2} \rho^{e} \left( {\alpha_{1}^{e2} - 2\alpha_{2}^{e2} \sin^{2} \theta_{2} } \right)}}{{\iota \omega \beta_{1} T_{0} }}$$, $$C_{T} = \frac{{c_{11} \beta^{e} }}{{\beta_{1} }}$$.

$$\Delta_{17} = - \frac{{k_{3}^{2} \rho^{e} \alpha_{1}^{e2} \sin 2\theta_{3} }}{{\iota \omega \beta_{1} T_{0} }}$$, $$\Delta_{25} = - \frac{{k_{1}^{2} \alpha_{1}^{e2} \rho^{e} \sin 2\theta_{1} }}{{\iota \omega \beta_{1} T_{0} }}$$, $$\Delta_{26} = - \frac{{k_{2}^{2} \alpha_{2}^{e2} \rho^{e} \sin 2\theta_{2} }}{{\iota \omega \beta_{1} T_{0} }}$$, $$\Delta_{27} = - \frac{{k_{3}^{2} \alpha_{2}^{e2} \rho^{e} \cos 2\theta_{3} }}{{\iota \omega \beta_{1} T_{0} }}$$, $$\Delta_{35} = \iota k_{1} \sin \theta_{1}$$, $$\Delta_{36} = \iota k_{2} \sin \theta_{2}$$, $$\Delta_{37} = \iota k_{3} \cos \theta_{3}$$, $$\Delta_{45} = - \iota k_{1} \cos \theta_{1}$$,

$$\Delta_{46} = - \iota k_{2} \cos \theta_{2}$$, $$\Delta_{47} = \iota k_{3} \sin \theta_{3}$$, $$\Delta_{55} = - \zeta_{1}$$, $$\Delta_{56} = - \zeta_{2}$$, $$\Delta_{65} = \frac{{ - \zeta_{1} k_{1} K_{e}^{*} \cos \theta_{1} }}{\omega }$$$$\Delta_{66} = \frac{{ - \zeta_{2} k_{2} \cos \theta_{2} K_{e}^{*} }}{\omega }$$, $$X_{i} = \frac{{H_{i} }}{{A^{*} }}\,\,\left( {i = 1 - 4} \right)\,,$$ $$X_{5} = \frac{{A_{1}^{e} }}{{A^{*} }}$$, $$X_{6} = \frac{{B_{1}^{e} }}{{A^{*} }}$$, $$X_{7} = \frac{{D_{1}^{e} }}{{A^{*} }}$$.

- For incidence *P* wave: $A^{*} = A_{0}^{e}$, $Q_{1} = - \Delta_{15}$, $Q_{2} = \Delta_{25}$, $Q_{3} = - \Delta_{35}$, $Q_{4} = \Delta_{45}$, $Q_{5} = - \Delta_{55}$, $Q_{6} = \Delta_{65}$.
- For incidence *T* wave: $A^{*} = B_{0}^{e}$, $Q_{1} = - \Delta_{16}$, $Q_{2} = \Delta_{26}$, $Q_{3} = - \Delta_{36}$, $Q_{4} = \Delta_{46}$, $Q_{5} = - \Delta_{56}$, $Q_{6} = \Delta_{66}$.
- For incidence *SV* wave: $A^{*} = D_{0}^{e}$, $Q_{1} = \Delta_{17}$, $Q_{2} = - \Delta_{27}$, $Q_{3} = \Delta_{37}$, $Q_{4} = - \Delta_{47}$, $Q_{5} = 0$, $Q_{6} = 0$.

## Energy ratios

The average energy flux of the incident, refracted, and reflected waves could be used to figure out the energy distribution between refracted and reflected waves at the interface $x_{3} = 0$, across a unit area of the surface element. According to Kumar and Sharma^45^, the normal acoustic flux in an HPS material is represented by as follows

45 $$ P = - {\text{Re}} \;\left( {\sigma_{13} \overline{{\dot{u}}}_{1} + \sigma_{33} \overline{{\dot{u}}}_{3} - \phi \overline{\dot{D}}_{3} + K_{3} \overline{T}_{3} ,\frac{T}{{T_{0} }}} \right), $$

and for the incident and reflected waves for the elastic phase are

46 $$ P^{e} = - {\text{Re}} \,\left( {\sigma_{31}^{e} \overline{{\dot{u}}}_{1}^{e} + \sigma_{33}^{e} \overline{{\dot{u}}}_{3}^{e} } \right). $$

The average energy fluxes of the incident waves are as follows

47 $$ \left\langle {P_{IP}^{e} } \right\rangle = \frac{1}{2}\left( {\alpha_{1}^{e2} \rho^{e} k_{1}^{2} + C_{T} \zeta_{1} } \right)k_{1} \omega {\text{Re}} \left( {\cos \theta_{0} } \right)\left| {A_{0}^{e} } \right|^{2} , $$

48 $$ \left\langle {P_{IT}^{e} } \right\rangle = \frac{1}{2}\left( {\alpha_{1}^{e2} \rho^{e} k_{2}^{2} + C_{T} \zeta_{2} } \right)k_{2} \omega {\text{Re}} \left( {\cos \theta_{0} } \right)\left| {B_{0}^{e} } \right|^{2} , $$

49 $$ \left\langle {P_{IS}^{e} } \right\rangle = \frac{1}{2}\alpha_{2}^{e2} \rho^{e} k_{3}^{3} \omega {\text{Re}} \left( {\cos \theta_{0} } \right)\left| {D_{0}^{e} } \right|^{2} . $$

The average energy fluxes of the reflected waves are as follows

50 $$ \left\langle {P_{RP}^{e} } \right\rangle = - \frac{1}{2}\left( {\alpha_{1}^{e2} \rho^{e} k_{1}^{2} + C_{T} \zeta_{1} } \right)\,k_{1} \omega {\text{Re}} \left( {\cos \theta_{1} } \right)\left| {A_{1}^{e} } \right|^{2} , $$

51 $$ \left\langle {P_{RT}^{e} } \right\rangle = - \frac{1}{2}\left( {\alpha_{1}^{e2} \rho^{e} k_{2}^{2} + C_{T} \zeta_{2} } \right)\,k_{2} \omega {\text{Re}} \left( {\cos \theta_{2} } \right)\left| {B_{1}^{e} } \right|^{2} , $$

52 $$ \left\langle {P_{RS}^{e} } \right\rangle = - \frac{1}{2}\alpha_{2}^{e2} \rho^{e} k_{3}^{3} \omega {\text{Re}} \left( {\cos \theta_{3} } \right)\left| {D_{1}^{e} } \right|^{2} . $$

The average energy fluxes of the refracted waves are as follows

53 $$ \left\langle {P_{s} } \right\rangle = \frac{1}{2}\omega^{2} {\text{Re}} \left( {\Delta_{2s} + \Delta_{1s} \overline{W}_{s} + \overline{\Delta }_{7s} \Phi_{s} + \frac{\iota }{\omega }\frac{{K_{3} }}{{T_{0} }}\overline{\Delta }_{5s} \Theta_{s} } \right)\,\left| {H_{s} } \right|^{2} ,\;(s = 1 - 4). $$

The energy ratios of the reflected and refracted waves are defined as.

(i) for incident $P$ wave

54 $$ E_{RP} = \frac{{\left\langle {P_{RP}^{e} } \right\rangle }}{{\left\langle {P_{IP}^{e} } \right\rangle }},\,E_{RT} = \frac{{\left\langle {P_{RT}^{e} } \right\rangle }}{{\left\langle {P_{IP}^{e} } \right\rangle }},\,E_{RS} = \frac{{\left\langle {P_{RS}^{e} } \right\rangle }}{{\left\langle {P_{IP}^{e} } \right\rangle }},\,ES_{s} = \frac{{\left\langle {P_{s} } \right\rangle }}{{\left\langle {P_{IP}^{e} } \right\rangle }},\,\,\,\,(s = 1 - 4). $$

(ii) for incident $T$ wave

55 $$ E_{RP} = \frac{{\left\langle {P_{RP}^{e} } \right\rangle }}{{\left\langle {P_{IT}^{e} } \right\rangle }},\,E_{RT} = \frac{{\left\langle {P_{RT}^{e} } \right\rangle }}{{\left\langle {P_{IT}^{e} } \right\rangle }},\,E_{RS} = \frac{{\left\langle {P_{RS}^{e} } \right\rangle }}{{\left\langle {P_{IT}^{e} } \right\rangle }},\,ES_{s} = \frac{{\left\langle {P_{s} } \right\rangle }}{{\left\langle {P_{IT}^{e} } \right\rangle }},\,\,\,\,(s = 1 - 4). $$

(iii) for incident $SV$ wave

56 $$ E_{RP} = \frac{{\left\langle {P_{RP}^{e} } \right\rangle }}{{\left\langle {P_{IS}^{e} } \right\rangle }},\,E_{RT} = \frac{{\left\langle {P_{RT}^{e} } \right\rangle }}{{\left\langle {P_{IS}^{e} } \right\rangle }},\,E_{RS} = \frac{{\left\langle {P_{RS}^{e} } \right\rangle }}{{\left\langle {P_{IS}^{e} } \right\rangle }},\,ES_{s} = \frac{{\left\langle {P_{s} } \right\rangle }}{{\left\langle {P_{IS}^{e} } \right\rangle }},\,\,\,\,(s = 1 - 4). $$

The interaction energy ratios (interaction between different fields and displacement corresponding to refracted waves) are.

$E_{st} = \frac{{\left\langle {P_{st} } \right\rangle }}{{\left\langle {P_{IP}^{e} } \right\rangle }}$ for an incident of $P$ wave, $E_{st} = \frac{{\left\langle {P_{st} } \right\rangle }}{{\left\langle {P_{IT}^{e} } \right\rangle }}$ for an incident of $T$ wave, and $E_{st} = \frac{{\left\langle {P_{st} } \right\rangle }}{{\left\langle {P_{IS}^{e} } \right\rangle }}$ for an incident of $SV$ wave.

Where

57 $$ \left\langle {P_{st} } \right\rangle = \frac{1}{2}\omega^{2} {\text{Re}} \left( {\Delta_{2s} H_{s} \overline{H}_{t} + \Delta_{1s} \overline{W}_{t} H_{s} \overline{H}_{t} + \overline{\Delta }_{7s} \Phi_{t} H_{t} \overline{H}_{s} + \frac{\iota }{\omega }\frac{{K_{3} }}{{T_{0} }}\overline{\Delta }_{5s} \Theta_{s} \overline{H}_{s} H_{t} } \right). $$

The energy is conserved if

58 $$ \sum\limits_{s = 1}^{4} {\left( {ES_{s} + E_{{\text{int}}} + E_{RP} + E_{RT} + E_{RS} } \right) = 1} , $$

where $E_{{\text{int}}} = \sum\limits_{s,t = 1,s \ne t}^{4} {E_{st} }$ is the resultant interaction energy between the refracted waves.

## Discussion and numerical findings

The energy ratios for the incidence longitudinal *P* wave, thermal *T* wave, or transversal *SV* wave at the interface of TS/HPS are computed and plotted graphically with the help of MatLab software for a particular model of TS medium (magnesium) and HPS medium (cadmium selenide). The material parameters of cadmium selenide and magnesium are borrowed from Mondal and Othman^46^ and Kumar and Sarthi^47^, as shown in Table 1.

**Table 1:** Values of the materials constants.

Symbol	Value	Symbol	Value
$c_{11}$	$74.1 \times 10^{9} \;{\text{Nm}}^{ - 2}$	$T_{0}$	$298\;{\text{K}}$
$c_{12}$	$45.2 \times 10^{9} \;{\text{Nm}}^{ - 2}$	$\beta_{1}$	$6.21 \times 10^{5} \;{\text{Nm}}^{ - 2} {\text{K}}^{ - 1}$
$c_{13}$	$39.3 \times 10^{9} \;{\text{Nm}}^{ - 2}$	$\beta_{3}$	$5.51 \times 10^{5} \;{\text{Nm}}^{ - 2} {\text{K}}^{ - 1}$
$c_{33}$	$83.6 \times 10^{9} \;{\text{Nm}}^{ - 2}$	$\eta_{13}$	$- 0.160\;{\text{Cm}}^{ - 2}$
$c_{55}$	$13.2 \times 10^{9} \;{\text{Nm}}^{ - 2}$	$\eta_{15}$	$- 0.138\;{\text{Cm}}^{ - 2}$
$\varepsilon_{11}$	$8.26 \times 10^{ - 11} \,{\text{C}}^{2} {\text{N}}^{ - 1} {\text{m}}^{ - 2}$	$\eta_{33}$	$0.347\;{\text{Cm}}^{ - 2}$
$\varepsilon_{33}$	$9.03 \times 10^{ - 11} \,{\text{C}}^{2} {\text{N}}^{ - 1} {\text{m}}^{ - 2}$	$C_{E}^{e}$	$1.04 \times 10^{3} \,{\text{Jkg}}^{ - 1} \deg^{ - 1}$
$p_{3}$	$- 2.9 \times 10^{ - 6} \,{\text{Cm}}^{ - 2} {\text{K}}^{ - 1}$	$\lambda^{e}$	$2.696 \times 10^{10} \;{\text{Nm}}^{ - 2}$
$K_{1} ,\,K_{3}$	$9\,{\text{Wm}}^{ - 1} {\text{k}}^{ - 1}$	$\mu^{e}$	$1.639 \times 10^{10} \;{\text{Nm}}^{ - 2}$
$K_{1}^{*} ,\,K_{3}^{*}$	$7\;{\text{Wm}}^{ - 1} {\text{k}}^{ - 1} {\text{s}}^{ - 1}$	$\rho^{e}$	$1.74 \times 10^{3} \;{\text{kgm}}^{ - 3}$
$K_{e}^{*}$	${{C_{E}^{e} (\lambda + 2\mu )} \mathord{\left/ {\vphantom {{C_{E}^{e} (\lambda + 2\mu )} 4}} \right. \kern-0pt} 4}$	$\beta^{e}$	$2.68 \times 10^{6} \;{\text{Nm}}^{ - 2} \deg^{ - 1}$
$\rho$	$5504\;{\text{kgm}}^{ - 3}$	$\omega$	$100\;{\text{Hz}}$

**Figure 2:**
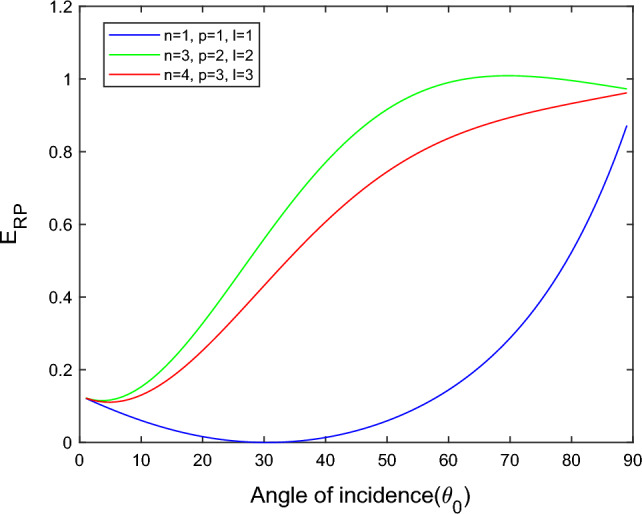
Variation of energy ratio $E_{RP}$ with $\theta_{0}$.

**Figure 3:**
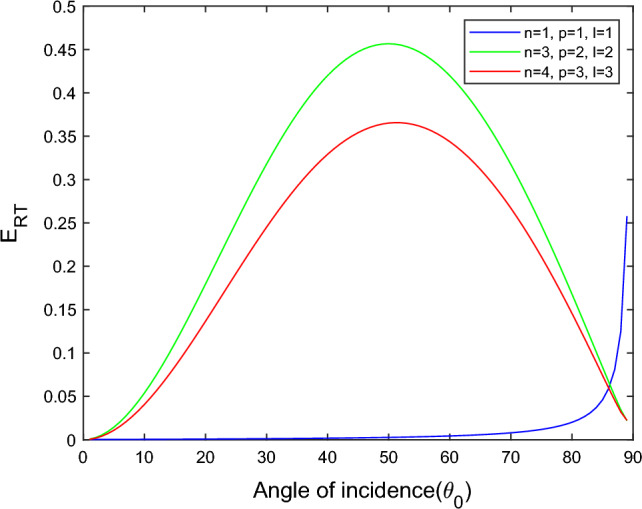
Variation of energy ratio $E_{RT}$ with $\theta_{0}$.

**Figure 4:**
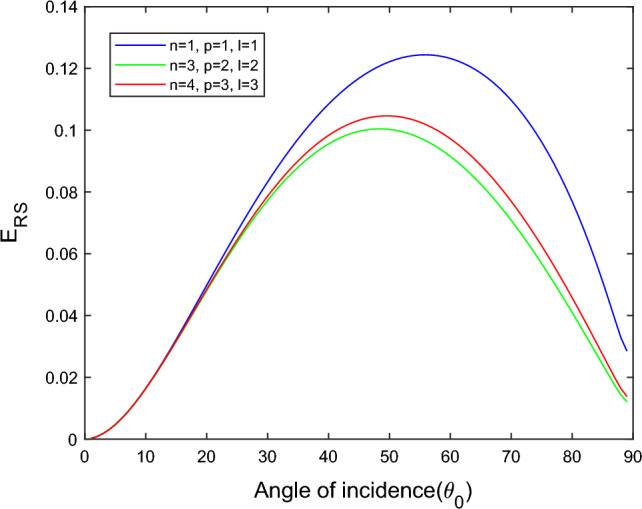
Variation of energy ratio $E_{RS}$ with $\theta_{0}$.

**Figure 5:**
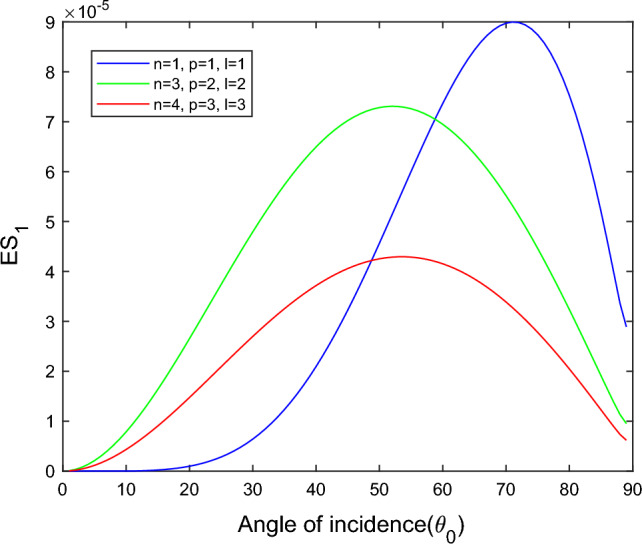
Variation of energy ratio $ES_{1}$ with $\theta_{0}$.

**Figure 6:**
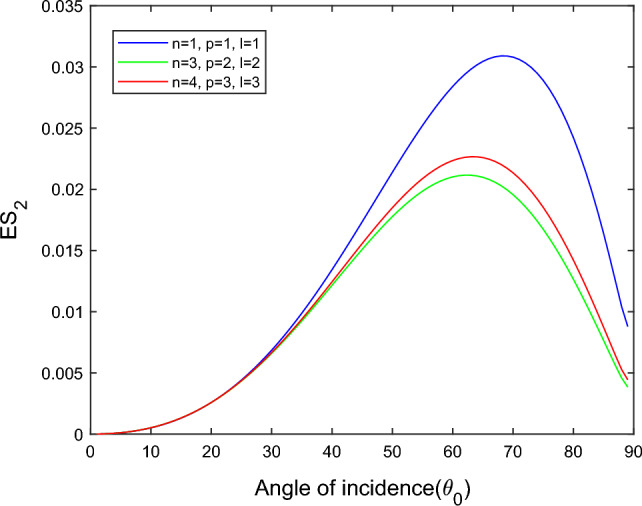
Variation of energy ratio $ES_{2}$ with $\theta_{0}$.

**Figure 7:**
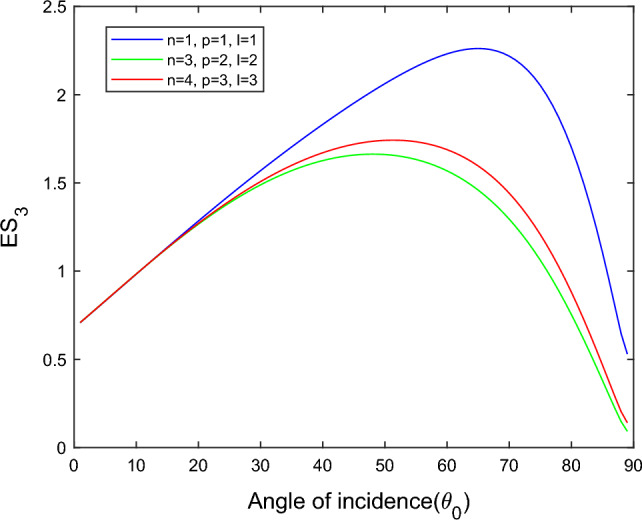
Variation of energy ratio $ES_{3}$ with $\theta_{0}$.

**Figure 8:**
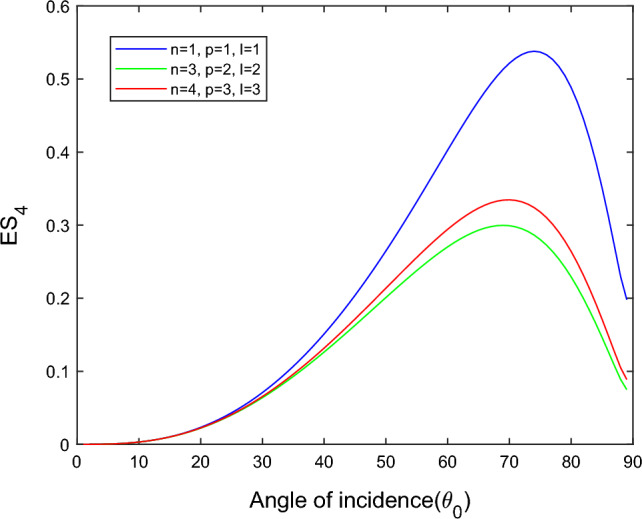
Variation of energy ratio $ES_{4}$ with $\theta_{0}$.

**Figure 9:**
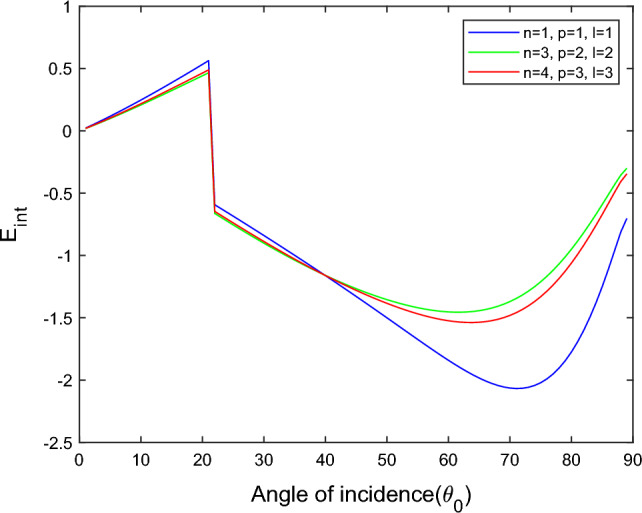
Variation of energy ratio $E_{{\text{int}}}$ with $\theta_{0}$.

**Figure 10:**
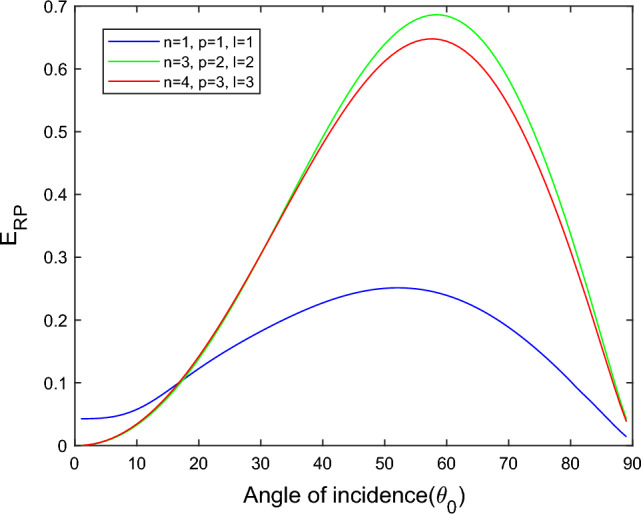
Variation of energy ratio $E_{RP}$ with $\theta_{0}$.

**Figure 11:**
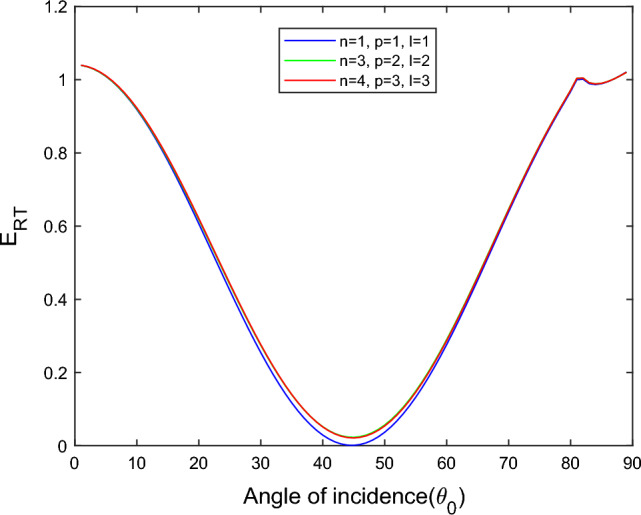
Variation of energy ratio $E_{RT}$ with $\theta_{0}$.

**Figure 12:**
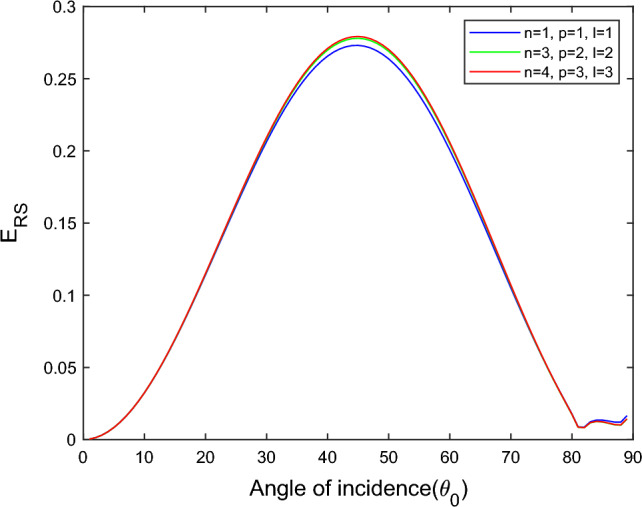
Variation of energy ratio $E_{RS}$ with $\theta_{0}$.

**Figure 13:**
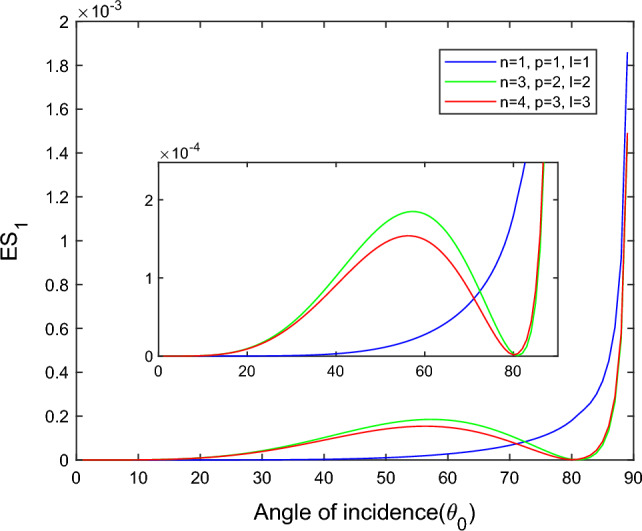
Variation of energy ratio $ES_{1}$ with $\theta_{0}$.

**Figure 14:**
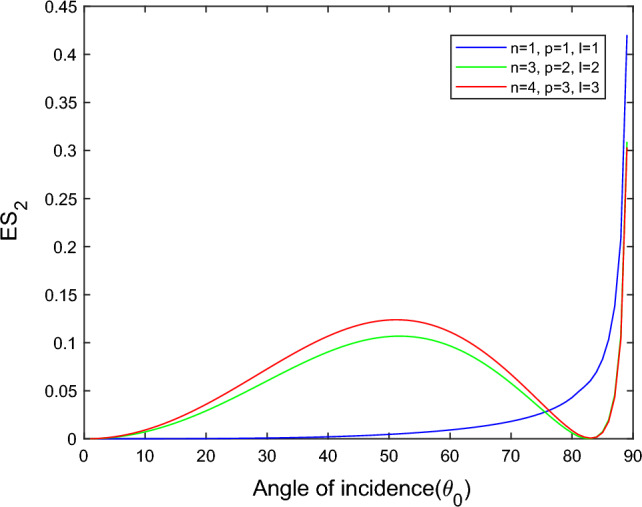
Variation of energy ratio $ES_{2}$ with $\theta_{0}$.

**Figure 15:**
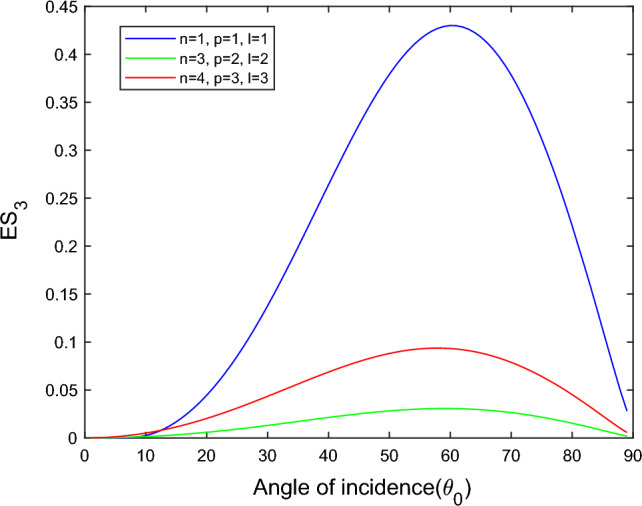
Variation of energy ratio $ES_{3}$ with $\theta_{0}$.

**Figure 16:**
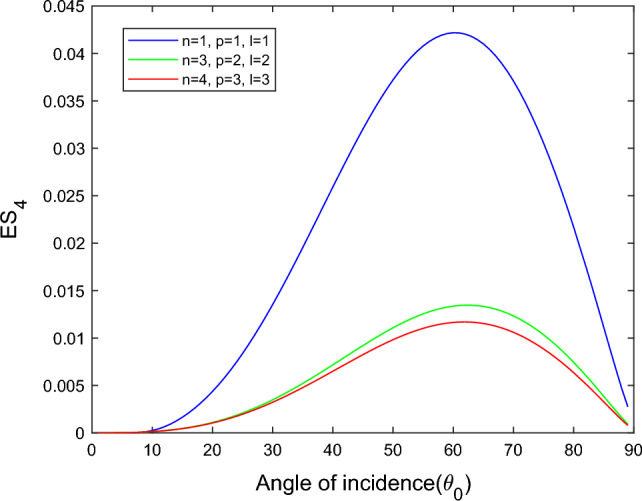
Variation of energy ratio $ES_{4}$ with $\theta_{0}$.

**Figure 17:**
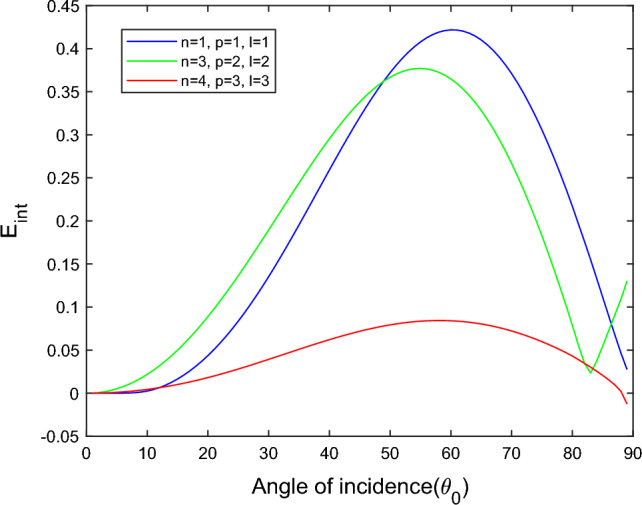
Variation of energy ratio $E_{{\text{int}}}$ with $\theta_{0}$.

**Figure 18:**
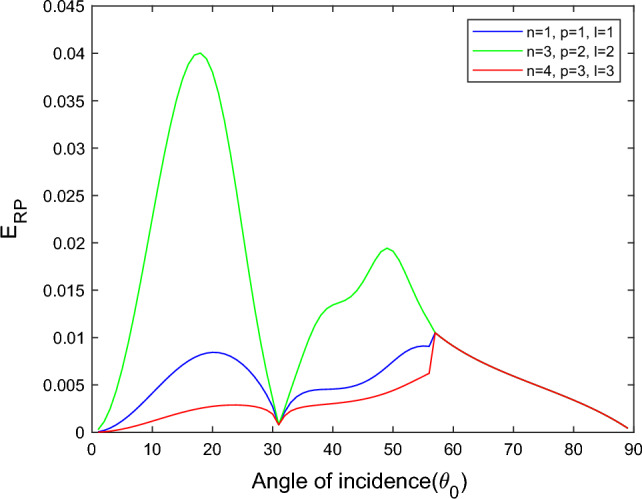
Variation of energy ratio $E_{RP}$ with $\theta_{0}$.

**Figure 19:**
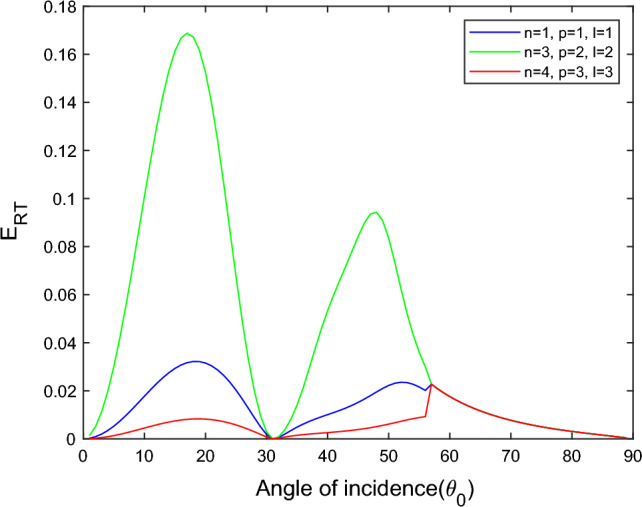
Variation of energy ratio $E_{RT}$ with $\theta_{0}$.

**Figure 20:**
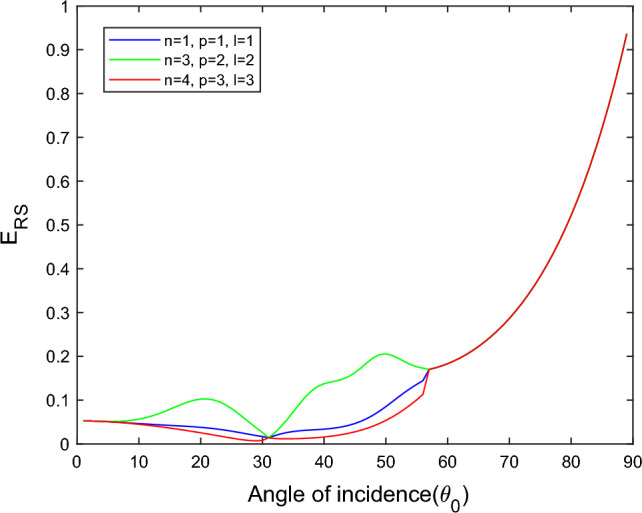
Variation of energy ratio $E_{RS}$ with $\theta_{0}$.

**Figure 21:**
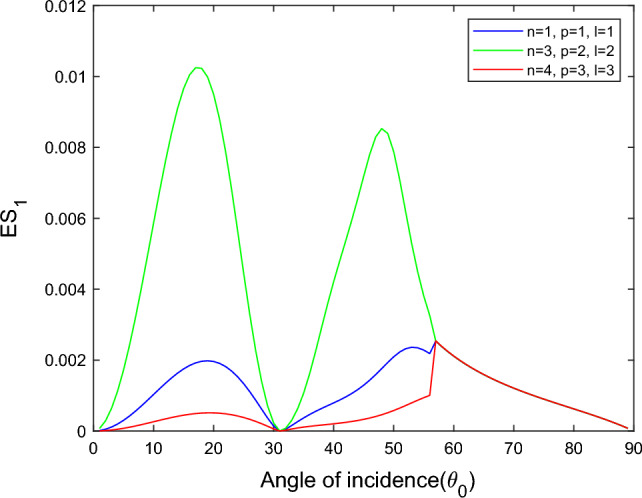
Variation of energy ratio $ES_{1}$ with $\theta_{0}$.

**Figure 22:**
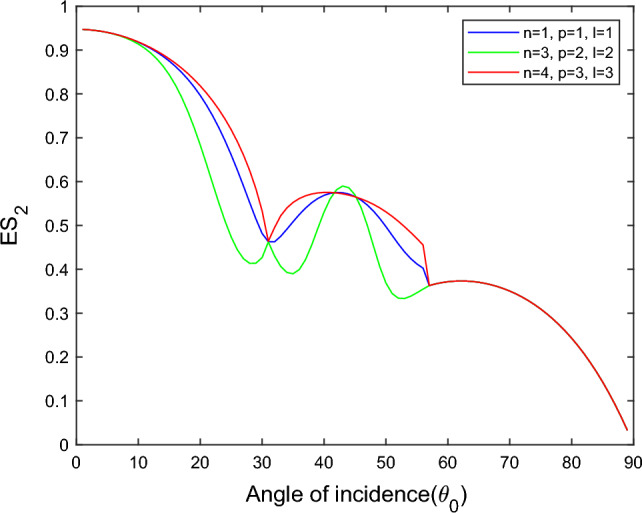
Variation of energy ratio $ES_{2}$ with $\theta_{0}$.

**Figure 23:**
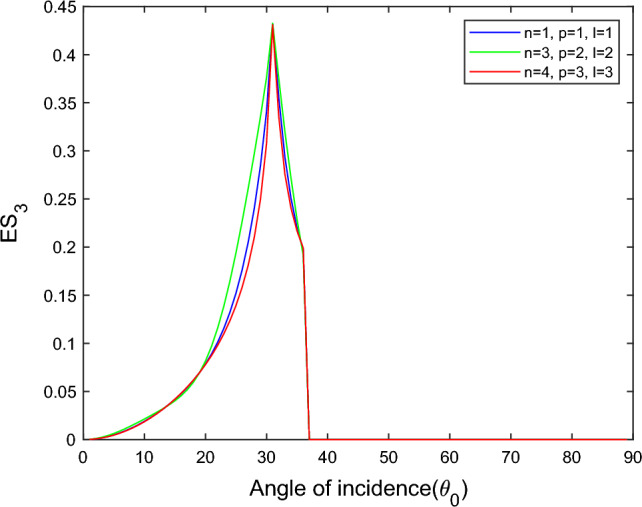
Variation of energy ratio $ES_{3}$ with $\theta_{0}$.

**Figure 24:**
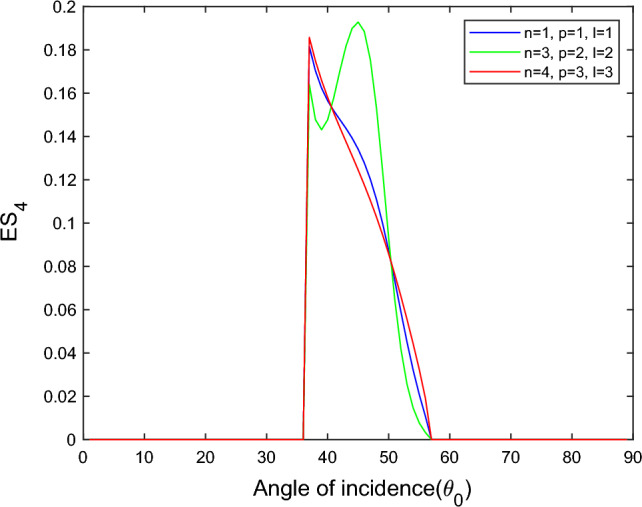
Variation of energy ratio $ES_{4}$ with $\theta_{0}$.

**Figure 25:**
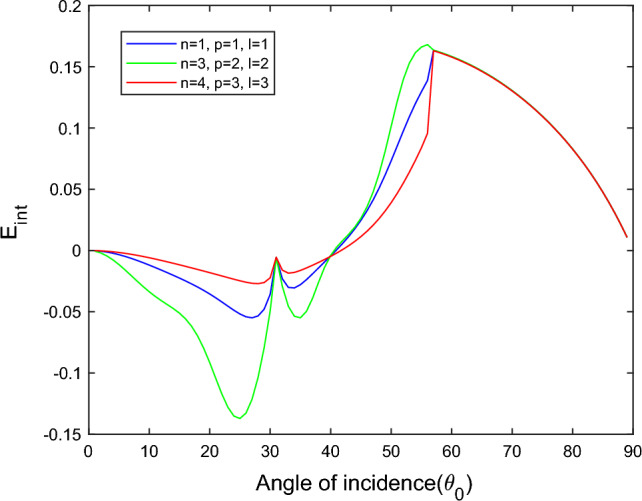
Variation of energy ratio $E_{{\text{int}}}$ with $\theta_{0}$.

The most notable benefit of this extended model is that it is based on heat transfer with MDD of order ($n,\,p,$ and $l$), and its flexibility in applications due to the free choice of the delay time factor and kernel function as stated earlier in Eqs. (1) and (2) Chiriţă et al.^38^ show that $n \ge 5$ or $p \ge 5$ leads to an unstable system and cannot accurately describe an actual situation. Zampoli^48^ states that the expansion orders must be less than or equal to 4 for the accompanying models compatible with thermodynamically provided that the correct phase lag time assumptions are made. We choose the kernel function $K_{i} \left( {t - \zeta } \right) = 1 - {{\left( {t - \zeta } \right)} \mathord{\left/ {\vphantom {{\left( {t - \zeta } \right)} \tau }} \right. \kern-0pt} \tau }_{i} ;\,$ $i = q,\,\,T,\,\,v$ and phase lags $\tau_{q} = 0.05\,s$, $\tau_{T} = 0.03s$, $\tau_{v} = 0.02s$ such that they fulfill the conditions established by Quintanilla and Racke^49^. To study the impact of higher-order MDD ($n,\,p,$ and $l$) on the variations of the energy ratios, we developed the three different models according to three distinct choices of $n$,$p$ and $l$ such that $n = 4$, $p = 3$, $l = 3$; $n = 3$, $p = 2$, $l = 2$; and $n = p = l = 1$ represented by solid red, green, and blue lines respectively as shown in the Figs. 2, 3, 4, 5, 6, 7, 8, 9, 10, 11, 12, 13, 14, 15, 16, 17, 18, 19, 20, 21, 22, 23, 24, 25. $E_{RP}$, $E_{RT}$, and $E_{RS}$ stand for energy ratios corresponding to reflected *P*, *T*, and *SV* waves, respectively, and $ES_{i}$; $i = 1 - 4$ stand for refracted *qP*, *qT*, *qS*, and *eP* waves, respectively. The overall interaction energy ratio between the various refracted waves is denoted by the $E_{{\text{int}}}$.

### For incident *P* wave

Figure 2 depicts that for the model $n = p = l = 1$, the magnitude of $E_{RP}$ is first decreases gradually with an increased angle of incidence $0^\circ \le \theta_{0} \le 30^\circ$. After a further increase in the angle of incidence $30^\circ \le \theta_{0} \le 90^\circ$, the magnitude of $E_{RP}$ increases monotonically. But in the $n = 4$, $p = 3$, $l = 3$ and $n = 3$, $p = 2$, $l = 2$ models, we noticed that magnitude of $E_{RP}$ increases with $\theta_{0}$, but the increment is slow at near the grazing and normal incidence. *P* wave reflects more in the $n = 3$, $p = 2$, $l = 2$ model than the other two considered models.

In Fig. 3, we observed that the magnitude of $E_{RT}$ increases with $\theta_{0}$ go to maximum and then decreases approaches to zero near the grazing incidence and follow the parabolic path for the $n = 4$, $p = 3$, $l = 3$ and $n = 3$, $p = 2$, $l = 2$ models. On the other hand, for the $n = p = l = 1$ model, the magnitude of $E_{RT}$ approaches zero in a complete range of considered $\theta_{0}$ but near the grazing incidence magnitude of $E_{RT}$ increases rapidly.

Figure 4 shows that the magnitude of $E_{RS}$ first increase with the angle of incidence goes to maximum and then decreases with a further increase of angle of incidence. The magnitude of reflected *SV* waves is dominated in the $n = p = l = 1$ model following the $n = 4$, $p = 3$, $l = 3$ and $n = 3$, $p = 2$, $l = 2$ models. In all considered models, fewer SV waves are reflected near the normal and grazing incidence.

Figures 5, 6, 7 and 8 reveal that the transmitted wave modes, namely, the qS mode, propagate more easily in a piezothermoelastic medium than the other modes. In contrast, *qP* mode is significantly less excited in the piezothermoelastic medium. Except for the *qS* mode, all transmitted modes are not excited near the normal incidence. The *qT*, *qS,* and *eP* wave modes are more excited for the $n = p = l = 1$ model compared to two other considered models in the complete range of angle of incidence. In contrast, initially, the *qP* wave mode is more enthusiastic in the $n = 3$, $p = 2$, $l = 2$ model but after $\theta_{0} > 58^\circ$ the $n = p = l = 1$ model dominates over the other two models.

From Fig. 9, we noticed that the $E_{{\text{int}}}$ increases with increases angle of incidence up to $\theta_{0} = 21^\circ$ after that, critical angle interaction energy ratios become positive to negative, and with further increases with angle of incidence, the interaction energy decreases, and at near the grazing incidence, interaction energy again increases.

### For incident *T* wave

In all three models taken into consideration, Fig. 10 demonstrates that the magnitude of $E_{RP}$ initial rises grows monotonically to a maximum and subsequently declines to a minimum, following a parabolic path with a rising angle of incidence. *P* waves reflected more readily in the $n = 4$, $p = 3$, $l = 3$ and $n = 3$, $p = 2$, $l = 2$ models than the $n = p = l = 1$ model.

Figure 11 shows that in all three considered models, the magnitude of $E_{RT}$ first monotonically falls approaches zero, with an angle of incidence $0^\circ \le \theta_{0} \le 45^\circ$, then monotonically grows and reaches unity with the angle of incidence $45^\circ \le \theta_{0} \le 90^\circ$. Higher order MDD parameters $n$, $p$, $l$ do not seem to have any discernible effects. Figure 12 demonstrates that the magnitude of $E_{RS}$ the whole range of angle of incidence follows the almost inverted pattern of Fig. 11.

Figures 13 and 14 reveal that as the angle of incidence increases, the magnitude of $ES_{1}$ and $ES_{2}$ increases gradually for the $n = p = l = 1$ model while for the $n = 4$, $p = 3$, $l = 3$ and $n = 3$, $p = 2$, $l = 2$ models follow the parabolic path whereas around grazing incidence, the magnitude of $ES_{1}$ and $ES_{2}$ climb extremely fast in all three considered models. The sub-indent figure of Fig. 13 depicts the magnified image of the overlapping curves to observe the variation in the microscale level. For the $n = p = l = 1$ model, the transmitted wave modes *qP* and *qT* propagate quickly near the grazing incidence compared to the other two models. While in the remaining range of angle of incidence, the magnitude of *qP* mode in the $n = 3$, $p = 2$, $l = 2$ model dominated over the $n = 4$, $p = 3$, $l = 3$ model and reversed behavior is observed in the *qT* mode*.*

The *qS* and *eP* wave modes transfer rapidly in the piezothermoelastic medium at the mid-angle of incidence, as shown in Figs. 15 and 16. On the other hand, *qS* and *eP* modes are less excited near the grazing incidence and are not excited near the normal incidence for all three considered models. The magnitude of *qS* wave mode dominates for the $n = p = l = 1$ model, followed by the $n = 4$, $p = 3$, $l = 3$ and $n = 3$ $p = 2$, $l = 2$ models. In contrast, the magnitude of *eP* wave mode dominates for the $n = p = l = 1$ model, followed by the $n = 4$, $p = 3$, $l = 3$ and $n = 3$, $p = 2$, $l = 2$ models.

Figure 17 shows that the incidence wave’s interaction energy ratio initially increases to the maximum and then decreases with the angle of incidence. For the $n = 4$, $p = 3$, $l = 3$ model, the magnitude of $E_{{\text{int}}}$ most minor compared to the other two models. In the $n = 3$, $p = 2$, $l = 2$ model near the grazing incidence, the magnitude of $E_{{\text{int}}}$ sharply increases, while in the other two models, it slightly decreases.

### For incident *SV* wave

Figures 18, 19 and 20 reveal that reflected energy ratios $\left| {E_{RP} } \right|$ and $\left| {E_{RT} } \right|$ in the range of angle of incidence $0^{0} \le \theta_{0} \le 58^{0}$ follows the two peaks in contrast $\left| {E_{RS} } \right|$ trend almost linear and follow the two peaks only for the $n = 3$, $p = 2$, $l = 2$ model. Figures 18 and 19 follow nearly the same pattern, but their magnitudes differ. After the $\theta_{0} = 58^\circ$, no significant impact of higher-order MDD parameters is observed. For the $n = 3$, $p = 2$, $l = 2$ model, the magnitude of reflected energy ratios is maximum, followed by $n = p = l = 1$ and $n = 4$, $p = 3,$ $l = 3$ models. But at near grazing incidence, the *qT* mode is no longer excited. After $\theta_{0} = 58^\circ$ all the curves overlap, the impact of higher-order parameters has disappeared.

Figures 21, 22, 23 and 24 depict that the magnitude of all transmitted wave modes corresponding to the $n = p = l = 1$ model lies between the $n = 4$, $p = 3$, $l = 3$ and $n = 3$, $p = 2$, $l = 2$ models. The *qP* and *qS* wave modes quickly propagate in piezothermoelastic medium for $n = 3$, $p = 2$, $l = 2$ model as compared to $n = p = l = 1$ and $n = 4$, $p = 3,$ $l = 3$ models. On the other hand, reverse behavior is observed for the propagation of the *qT* wave mode. The *qS* waves have a critical angle $\theta_{0} = 37^\circ$. After reaching this critical angle, the qS modes are no longer excited, and the impact of higher-order MDD parameters are disappeared since all curves overlap. As shown in Fig. 24, the electric potential wave does not propagate in a piezothermoelastic material for all investigated models except for the range of angle of incidence $36^\circ \le \theta_{0} \le 56^\circ$. The *eP* wave modes are highly stimulated at the angle of incidence $36^\circ \le \theta_{0} \le 45^\circ$.

Figure 25 illustrates the oscillating and almost reverse pattern seen in Figs. 18, 19, 20 and 21 for interaction energy ratios. The interaction energy changes from negative to positive at an angle of incidence $\theta_{0} = 40^\circ$. After $\theta_{0} \ge 58^\circ$, all four models’ curves coincide, as discussed in Figs. 18, 19, 20, 21 and 22. In the case of incidence *SV* wave as contrary to incidence *P* or *T* wave, for all energy ratios, a critical angle $\theta_{0} = 58^\circ$ is observed in all considered higher-order MDD models. The identification of a critical angle for the incidence of SV waves agrees with the study conducted by Barak et al.^17,20^.

## Conclusion

The thermoelastic plane wave phenomena at an interface between TS and HPS are examined in this study, and the effect of higher-order time differential parameters on energy ratios is studied. The energy ratios of various refracted and reflected waves are calculated using the amplitude ratios for incident P, T, or SV waves. We built three distinct models to investigate the effect of higher-order MDD ($n$, $p$, $l$) on the variation of the energy ratios according to three different choices of $n$, $p$, $l$ such that $n = 4$, $p = 3$, $l = 3$; $n = 3$, $p = 2$, $l = 2$; and $n = p = l = 1$. Following are some of the findings gleaned from this investigation:

- The energy ratios are influenced by factors such as the characteristics of the incident wave, higher-order MDD parameters, the angle of incidence, and the material’s physical properties. The nature of this reliance varies for various waves, as seen in Figs. 2, 3, 4, 5, 6, 7, 8, 9, 10, 11, 12, 13, 14, 15, 16, 17, 18, 19, 20, 21, 22, 23, 24, 25.
- For incidence *P* wave, *qS* wave mode is highly excited. It easily propagates in the piezothermoelastic medium compared to other transmitted waves, and the magnitude of the reflected *P* wave is maximum compared to other *T* or *SV* waves. The magnitude of all energy ratios for $n = 4$, $p = 3$, $l = 3$ model lies between the $n = p = l = 1$ and $n = 3$, $p = 2$, $l = 2$ models.
- For incidence *T* wave, *qP* and *qT* wave modes propagate in piezothermoelastic medium only near the grazing incidence. In contrast, *qS* and *eP* wave modes propagate in a mid-angle range of incidence. The negligible impact of higher-order MDD parameters is observed in reflected energy ratios of *T* and *SV* waves. In contrast, in other energy ratios, the effect varies with the angle of incidence.
- For incidence *SV* wave, near the normal incidence *qS* wave mode is highly excited and easily propagates in piezothermoelastic medium compared to other transmitted waves. After a particular angle $\theta_{0} = 58^{0}$, all energy ratios are independent of higher-order MDD parameters, i.e., all three curves overlap.
- It is discovered that, in all models considered, the total of the energy ratios is almost equal to one at each angle of incidence $0^\circ \le \theta_{0} \le 90^\circ$. As a result, each model supports the law of energy balance.

### Supplementary Information

Supplementary Information.

## Data Availability

All data generated or analyzed during this study are included in this published article [and its supplementary information file].
