# Supplementary material for: Behavior of higher-order MDD on energy ratios at the interface of thermoelastic and piezothermoelastic mediums
Source: Sci Rep. 2023 Oct 11;13:17170. doi: 10.1038/s41598-023-44339-5 (PMC10567774; doi:10.1038/s41598-023-44339-5)
Supplement: Supplementary file 1 — Supplementary Information. [file 41598_2023_44339_MOESM1_ESM.docx]

Legend Details

$E_{RP}$, $E_{RT}$, and $E_{RS}$ stand for energy ratios corresponding to reflected *P*, *T*, and *SV* waves, respectively, and $ES_{i}$; $i=1-4$ stand for refracted *qP*, *qT*, *qS*, and *eP* waves, respectively. The overall interaction energy ratio between the various refracted waves is denoted by the $E_{\mathrm{int}}$.

Appendix

The coefficients utilized in equation (4.4) are presented herein

$$G_{11}=l_{12}l_{22}l_{34}l_{42}+l_{12}l_{23}l_{34}l_{41}$$

$$\begin{matrix} G_{12}=cl_{14}l_{22}l_{34}-cl_{13}l_{23}l_{34}-cl_{13}l_{34}l_{42}-cl_{14}l_{34}l_{41}+d_{41}l_{12}l_{23}l_{34}+d_{24}l_{12}l_{34}l_{41}+d_{43}l_{12}l_{22}l_{34}+l_{11}l_{22}l_{34}l_{42} \\ l_{11}l_{23}l_{34}l_{41}+l_{12}l_{21}l_{34}l_{42}-l_{12}l_{22}l_{32}l_{43}+l_{12}l_{22}l_{33}l_{42}-l_{12}l_{23}l_{31}l_{43}+l_{12}l_{23}l_{33}l_{41}+l_{12}l_{24}l_{31}l_{42}-l_{12}l_{24}l_{32}l_{41} \end{matrix}$$

$$\begin{matrix} G_{13}=d_{24}d_{41}l_{12}l_{34}-cd_{41}l_{14}l_{34}-cd_{43}l_{13}l_{34}-cd_{24}l_{13}l_{34}-cl_{13}l_{23}l_{33}+cl_{13}l_{24}l_{32}+cl_{14}l_{21}l_{34}+cl_{14}l_{22}l_{33}+ \\ cl_{14}l_{24}l_{31}-cl_{15}l_{22}l_{32}-cl_{15}l_{23}l_{31}+cl_{13}l_{23}l_{43}-cl_{13}l_{24}l_{42}-cl_{14}l_{22}l_{43}-cl_{14}l_{24}l_{41}+cl_{15}l_{22}l_{42}+cm_{15}l_{23} \\ l_{41}+cl_{13}l_{32}l_{43}-cl_{13}l_{33}l_{42}+cl_{14}l_{31}l_{43}-cl_{14}l_{33}l_{41}-cl_{15}l_{31}l_{42}+cl_{15}l_{32}l_{41}+d_{41}l_{11}l_{23}l_{34}+d_{41}l_{12}l_{23}l_{33} \\ -d_{41}l_{12}l_{24}l_{32}+d_{24}l_{11}l_{34}l_{41}-d_{24}l_{12}l_{31}l_{43}+d_{24}l_{12}l_{33}l_{41}+d_{43}l_{11}l_{22}l_{34}+d_{43}l_{12}l_{21}l_{34}+d_{43}l_{12}l_{22}l_{33}+ \\ d_{43}l_{12}l_{24}l_{31}+l_{11}l_{21}l_{34}l_{42}-l_{11}l_{22}l_{32}l_{43}+l_{11}l_{22}l_{33}l_{42}-l_{11}l_{23}l_{31}l_{43}+l_{11}l_{23}l_{33}l_{41}+l_{11}l_{24}l_{31}l_{42}-l_{11}l_{24}l_{32}l_{41} \\ -l_{12}l_{21}l_{32}l_{43}+l_{12}l_{21}l_{33}l_{42} \end{matrix}$$

$$\begin{matrix} G_{14}=cd_{41}l_{15}l_{23}-cd_{24}l_{15}l_{31}-cd_{41}l_{14}l_{24}-cd_{24}l_{13}l_{33}+cd_{24}l_{13}l_{43}+cd_{24}l_{15}l_{41}-cd_{43}l_{13}l_{24}+cd_{43}l_{15}l_{22} \\ -cd_{41}l_{14}l_{33}+cd_{41}l_{15}l_{32}-cd_{43}l_{13}l_{33}-cd_{43}l_{15}l_{31}+d_{24}d_{41}l_{11}l_{34}+d_{24}d_{41}l_{12}l_{33}+cl_{14}l_{21}l_{33}-cl_{15}l_{21} \\ l_{32}-cl_{14}l_{21}l_{43}+cl_{15}l_{21}l_{42}+d_{41}l_{11}l_{23}l_{33}-d_{41}l_{11}l_{24}l_{32}-d_{24}l_{11}l_{31}l_{43}+d_{24}l_{11}l_{33}l_{43}+d_{24}l_{11}l_{33}l_{41}+d_{43} \\ l_{11}l_{21}l_{34}+d_{43}l_{11}l_{22}l_{33}+d_{43}l_{11}l_{24}l_{31}+d_{43}l_{12}l_{21}l_{33}-l_{11}l_{21}l_{32}l_{43}+l_{11}l_{21}l_{33}l_{42} \end{matrix}$$

$$G_{15}=cd_{43}l_{15}l_{21}+d_{24}l_{41}l_{11}l_{33}+d_{43}l_{11}l_{21}l_{33}+cd_{24}l_{41}l_{15}$$
